# Synthesis, Antibacterial and Antifungal Activity of New 3-Aryl-5*H*-pyrrolo[1,2-*a*]imidazole and 5*H*-Imidazo[1,2-*a*]azepine Quaternary Salts

**DOI:** 10.3390/molecules26144253

**Published:** 2021-07-13

**Authors:** Sergii Demchenko, Roman Lesyk, Oleh Yadlovskyi, Johannes Zuegg, Alysha G. Elliott, Iryna Drapak, Yuliia Fedchenkova, Zinaida Suvorova, Anatolii Demchenko

**Affiliations:** 1 Institute of Pharmacology and Toxicology, Antona Tsedika 14, 03057 Kyiv, Ukraine; demcha.chem@gmail.com (S.D.); yadlovskyi@online.ua (O.Y.); suvorovazina@gmail.com (Z.S.); demch7758@ukr.net (A.D.); 2 Department of Public Health, Dietetics and Lifestyle Disorders, Faculty of Medicine, University of Information Technology and Management in Rzeszow, 35-225 Rzeszow, Poland; 3 The Community for Open Antimicrobial Drug Discovery (CO-ADD), Centre for Superbug Solutions, Institute for Molecular Bioscience, The University of Queensland, Brisbane 4072, Australia; j.zuegg@imb.uq.edu.au (J.Z.); a.elliott@imb.uq.edu.au (A.G.E.); 4 Department of General, Inorganic, Physical and Colloidal Chemistry, Danylo Halytsky Lviv National Medical University, Pekarska 69, 79010 Lviv, Ukraine; iradrapak@ukr.net; 5 Department of Chemistry and Pharmacy, Nizhyn Mykola Gogol State University, 16600 Nizhyn, Ukraine; fja_fja@ukr.net

**Keywords:** 5*H*-pyrrolo[1,2-*a*]imidazoles, 5*H*-imidazo[1,2-*a*]azepines, quaternary salts, antibacterial activity, antifungal activity, in vitro tests, minimum inhibitory concentration

## Abstract

A series of novel 3-aryl-5*H*-pyrrolo[1,2-*a*]imidazole and 5*H*-imidazo[1,2-*a*]azepine quaternary salts were synthesized in 58–85% yields via the reaction of 3-aryl-6, 7-dihydro-5*H*-pyrrolo[1,2-*a*]imidazoles or 3-aryl-6,7,8,9-tetrahydro-5*H*-imidazo[1,2-*a*]azepines and various alkylating reagents. All compounds were characterized by ^1^H NMR, ^13^C NMR, and LC-MS. The conducted screening studies of the in vitro antimicrobial activity of the new quaternary salts derivatives established that 15 of the 18 newly synthesized compounds show antibacterial and antifungal activity. Synthesized 3-(3,4-dichlorohenyl)-1-[(4-phenoxyphenylcarbamoyl)-methyl]-6,7-dihydro-5*H*-pyrrolo[1,2-*a*]imidazol-1-ium chloride **6c** possessed a broad activity spectrum towards *Staphylococcus aureus*, *Escherichia coli*, *Klebsiella pneumoniae*, *Acinetobacter baumannii*, and *Cryptococcus neoformans,* with a high hemolytic activity against human red blood cells and cytotoxicity against *HEK-293*. However, compound **6c** is characterized by a low in vivo toxicity in mice (LD_50_ > 2000 mg/kg).

## 1. Introduction

Fighting infections has been one of the most pressing problems for mankind throughout its history. Infectious diseases occupy a leading place among the general morbidity of the population. The number of pathologies of infectious origin not only does not decrease, but also tends to increase. Insufficient effectiveness of antibiotic therapy is one of the main causes of mortality in patients with infectious diseases. For example, at least 50,000 people in Europe and the United States alone, and hundreds of thousands of people in other countries, die each year from infections caused by antimicrobial-resistant microorganisms [1].

Bacteria such as ESBL (extended spectrum β-lactamase)-producing *Enterobacteriaceae*, methicillin-resistant *Staphylococcus aureus* (MRSA), and vancomycin-resistant enterococci (VRE) are especially dangerous today, as these pathogens are resistant to most modern antimicrobial drugs. Thus, a study conducted in various countries determined the sensitivity of *Escherichia coli* to antibiotics and found that the level of resistance to 3rd generation cephalosporins and fluoroquinolones is significant and ranges between 68–95% and 48–89%, respectively, depending on the region [2,3].

In recent years, the number of mycoses caused by yeasts, dermatomycetes, and micromycetes has been increasing. Among these pathogens strains resistant to modern antifungal drugs are also found [4]. Thus, 76.9% and 82.2% of *Candida albicans* strains are insensitive to ketoconazole and fluconazole [5]. The frequency of isolation of *Cryptococcus neoformans* strains resistant to fluconazole is also increasing [6].

The increase in the number of infectious and infectious-inflammatory diseases is facilitated by the weakening of human immune status as a result of adverse environmental factors, uncontrolled use of antimicrobial drugs, and the use of cytostatics, to name a few [7]. The formation of pathogens resistant to antibiotics and the intensive spread of multidrug-resistant strains contribute to the reduction of antibiotic efficacy, which indicates the need for the continued discovery of novel active compounds and the creation of highly effective and safe antimicrobial drugs [8,9,10]. The search for such compounds is carried out among natural and synthetic substances.

**Figure 1:**
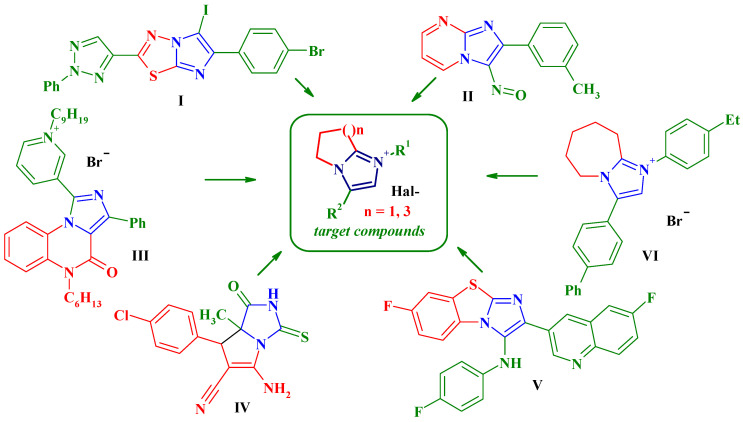
Background of the target compounds design.

Imidazole and its derivatives are of special interest as pharmacologically attractive heterocycles in medicinal chemistry. These compounds exhibit a widespread spectrum of biological activities. It is important to note that their antimicrobial and antifungal activities are some of the priorities for this class of heterocycles [11]. On the other hand, the combination of the imidazole ring with other heterocyclic moieties in condensed heterocyclic systems is one of the successful directions in the design of new molecules with antibacterial and antifungal activity. Thus, compounds with these types of activity have been identified among imidazo[2,1-*b*]-1,3,4-thiadiazoles **I** [12], imidazo[1,2-*a*]pyrimidines **II** [13], imidazo[1,5-*a*]quinoxalines **III** [14], pyrrolo[1,2-*c*]imidazoles **IV** [15], imidazo[2,1-*b*] [1,3] benzothiazoles **V** [16], etc. (Figure 1). Moreover, earlier we established the antifungal activity of derivatives of 3-aryl-6,7-dihydro-5*H*-pyrrolo[1,2-*a*]imidazole derivatives, structurally related to heterocycles [17]. Thus, quaternary salt **VI** (3-biphenyl-4-yl-1-(4-ethylphenyl)-6,7,8,9-tetrahydro-5*H*-imidazo[1,2-*a*]azepin-1-ium bromide) showed a polyvalent activity to *S. aureus*, *C. neoformans*, and *C. albicans* approaching reference drugs in their activity [18]. Given the above arguments, the aim of this work was to design novel 5*H*-pyrrolo[1,2-*a*]imidazole and 5*H*-imidazo[1,2-*a*]azepine quaternary salts as potential antimicrobial agents (Figure 1).

## 2. Results and Discussion

### 2.1. Chemistry

**Scheme 1:**
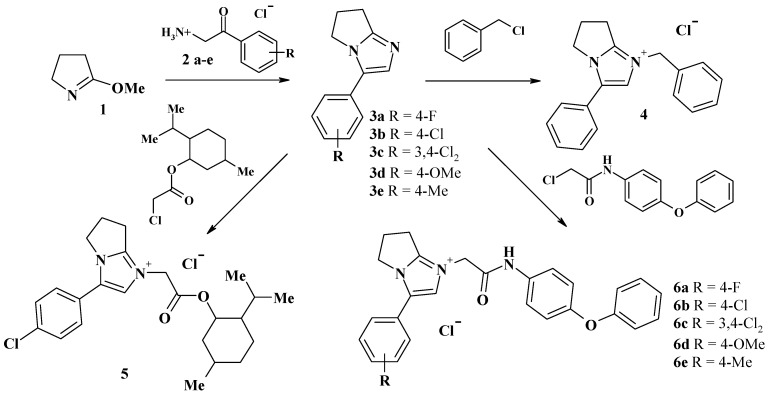
Synthetic routes for the preparation of compounds **4**, **5,** and **6a**–**e**.

**Scheme 2:**
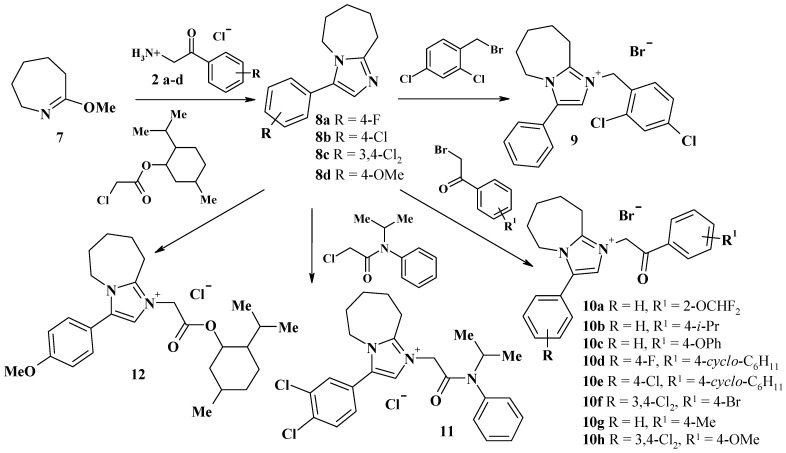
Synthetic routes for the preparation of compounds **8**, **9**, **10a**–**h**, **11,** and **12**.

1-Substituted-3-aryl-6,7-dihydro-5*H*-pyrrolo[1,2-*a*]imidazol-1-ium chlorides **4**, **5**, **6a**–**e** and 1-substituted-3-aryl-6,7,8,9-tetrahydro-5*H*-imidazo[1,2-*a*]azepin-1-ium chlorides or bromides **8**, **9**, **10a**–**h**, **11**, **12** were synthesized by Scheme 1 and Scheme 2.

3-Aryl-6,7-dihydro-5*H*-pyrrolo[1,2-*a*]imidazoles **3a**–**e** and 3-aryl-6,7,8,9-tetrahydro-5*H*-imidazo[1,2-*a*]azepines **8a**–**d** were obtained through the condensation of 5-methoxy-3,4-dihydro-2*H*-pyrrole **1** or 7-methoxy-3,4,5,6-tetrahydro-2*H*-azepine **7** with 2-amino-1-arylethanone **2a**–**e** using a well-known technique [19]. 2-Bromo-1-(2-difluoromethoxyphenyl)-ethanone (reagent for synthesis of **10a**) was synthesized using a known method [20]. Reaction of 3-aryl-6,7-dihydro-5*H*-pyrrolo[1,2-*a*]imidazoles **3a**–**e** and 3-aryl-6,7,8,9-tetrahydro-5*H*-imidazo[1,2-a]azepines **8a**–**d** with alkylating reagents leads to the formation of quaternary imidazolium salts **4**–**6** and **9**–**12**.

**Scheme 3:**
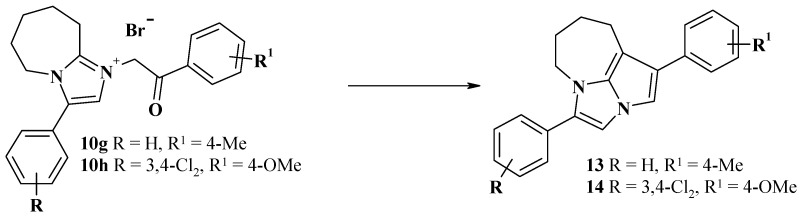
Synthetic routes for the preparation of compounds **13** and **14**.

Earlier, it was shown [21] that the heating of phenacyl salts **10** in an alkaline medium is accompanied by intramolecular condensation of the carbonyl group of the phenacyl fragment and the methylene group in the 9th position of the core heterocyclic system with the formation of 1,4-diaryl-5,6,7,8-tetrahydro-2a,4a-diazacyclopenta[*cd*]azulene (Scheme 3). This reaction is a logical continuation of the method for the synthesis of 2-phenylindolysine from 2-methylpyridine and phenacyl bromide proposed by A. Tschitschibabin in 1927 [22]. Based on this approach, derivatives **13** and **14** were obtained for the purpose of preliminary SAR analysis (Scheme 3).

The synthesized novel heterocyclic compounds were characterized by ^1^H and ^13^C NMR (Experimental part, Supplementary Materials). In the ^1^H NMR spectra of 3-aryl-5*H*-pyrrolo[1,2-*a*]imidazole quaternary salts **6a**–**e**, the signals of the saturated pyrrole ring were observed at 2.75–4.57 ppm. The two-proton singlet of the methylene group N^+^CH_2_CO for all compounds appears at 5.24–5.26 ppm. The one-proton singlet of the imidazole 2-CH-group was registered at 7.98–8.24 ppm. It should be noted that the position of this signal depends on the electronic properties of the benzene ring substituents in the third position of core heterocycle. Thus, for compound **6d** with an electron-donating group OMe, a single-proton singlet 2-CH was observed at 7.98 ppm. Whereas, in compound **6b,** with an electron withdrawing substituent Cl, the mentioned signal was appeared at 8.15 ppm. The introduction of the second chlorine atom into the third position of the benzene ring (compound **6c**) led to the appearance of the imidazole 2-CH proton in an even weaker field at 8.24 ppm. The methylene group N^+^CH_2_COO of menthol substituted compound **5** appeared as two doublets of the characteristic AB-system, at 5.26 and 5.36 ppm with *J* = 17.9 Hz, instead of the expected two-proton singlet. A similar picture was observed for the 3-(4-methoxyphenyl)-6,7,8,9-tetrahydro-5*H*-imidazo[1,2-*a*]azepine **15**. For this compound, the methylene group N^+^CH_2_COO was also registered as two doublets at 5.37 and 5.49 ppm with *J* = 18.1 Hz. This fact could be explained by the use of optically pure isomer *L*-menthol for the synthetic procedure.

In the ^1^H NMR spectra of 3-aryl-6,7,8,9-tetrahydro-5*H*-imidazo[1,2-*a*]azepines quaternary salts **9**–**12**, the signals of the azepine methylene groups appeared at 1.70–4.23 ppm. The two-proton singlet of the methylene group N^+^CH_2_CO phenacyl substituted 5*H*-imidazo[1,2-*a*]azepines **10b**–**f** was observed at 6.14–6.17 ppm. Whereas, for 2-difluoromethoxyphenyl derivative **10a** this signal was shifted to a higher field, at 5.95 ppm. This fact can be explained by the spatial screening of the methylene group by the difluoromethoxy group in the *ortho*-position of the phenacyl fragment. It should be noted that the signal of the OCHF_2_ group for compound **10a** was registered as a triplet at 7.43 ppm with *J* = 72.9 Hz. In the ^13^C NMR spectrum it was also observed as a triplet at 116.4 ppm with *J* = 259.2 Hz. The signal of the 2-CH-fragment of the imidazole ring for 5*H*-imidazo[1,2-*a*]azepines **9**–**12** was registered as a singlet in a narrow range at 7.69–7.77 ppm and practically does not depend on the electronic character of the substituents of the benzene ring in the third position of the core heterocycle.

In the ^1^H NMR spectrum of diazacyclopenta[*cd*]azulene **14**, the protons of the 6- and 7-CH_2_ groups correspond to two multiplets at 1.86 and 2.01 ppm, and the signals of the 8- and 5-CH_2_ groups were observed at 2.75 and 3.70 ppm, respectively. The singlets of the 2-CH pyrrole and 3-CH imidazole fragments were recorded at 6.80 and 7.39 ppm, respectively.

### 2.2. Biological Activity

Throughout the years of the antibiotic era, more than 1000 antimicrobial drugs have been discovered, of which only about 45 are used in clinical practice, with 6500 names. The cause of this situation is an irreversible biological phenomenon; the resistance of microorganisms. The overuse and misuse of antibiotics were highlighted as major contributing factors to this resistance [23]. Antibiotic resistance is associated with the high adaptive capacity of microbes and has existed since the beginning of the antibiotic era and is progressively increasing. Today, the main task of scientists is to find ways to overcome this. One of them is the search for new compounds with antimicrobial action for further development as new antimicrobial drugs. Novel 5*H*-pyrrolo[1,2-*a*]imidazole and 5*H*-imidazo[1,2-*a*]azepine quaternary salts are promising in this regard. These derivatives belong to the so-called quaternary ammonium compounds (QACs). QACs are a well-known class of cationic biocides with a broad spectrum of antimicrobial activity. QACs kill bacteria by impairing the permeability of cell membranes and do not contribute to antimicrobial resistance [24].

Assessment of biological activity was performed by the Community for Open Antimicrobial Drug Discovery (CO-ADD) [25,26], by testing the compounds against a range of pathogenic Gram-negative and Gram-positive bacteria, and fungi (yeasts).

The results of the biological studies indicated (Table 1) that all the tested heterocyclic compounds displayed activity against Gram-positive *S. aureus*; displaying a minimum inhibitory concentration (MIC) in a range between 2 and 32 μg/mL. Compounds **6b**, **6c**, **10c**–**e,** and **13** were the most active, with MIC values of 2–4 μg/mL (3.8–12.3 μM). The antistaphylococcal activity of 1-substituted-3-aryl-6,7-dihydro-5*H*-pyrrolo[1,2-*a*]imidazol-1-ium chlorides **6a**–**e** depends on the nature of the substitution of the phenyl moiety in position 3 of the core heterocycle. Thus, the presence of chlorine atoms in *para* or *meta* and *para* positions (compounds **6b**, **6c**) contributes to the highest effect level (MIC = 4 μg/mL/~8.0 μM). Replacement of the chlorine atom with a fluorine and methyl in position 4 of the benzene ring led to compounds **6a** and **6e** being two times less active than the previous two (**6b** and **6c**). The influence of the 4-OCH_3_ group (**6d**) further decreased the activity (MIC = 8 μg/mL). For 3-aryl-6,7,8,9-tetrahydro-5*H*-imidazo[1,2-*a*]azepin-1-ium chlorides **10b**–**e**, there is a certain relationship between the anti-staphylococcal activity and the character of substitution in the phenacyl moiety at position 1 of the core heterocycle. Thus, the most optimal are compounds with 4-cyclohexyl and 4-Cl (**10e**) and 4-cyclohexyl and 4-F (**10d**) and a combination of cyclohexyl and phenoxy (**10c**) substituents of two aromatic rings (MIC = 2–4 μg/mL/3.8–8.0 μM). The introduction of 3,4-di-Cl_2_ substitution in combination with 4-Br (**10f**) decreased the activity by four times, while the presence of isopropyl group was detrimental, leading to the less active compound, **10b** (MIC = 32 μg/mL/70.6 μM). On the other hand, replacement of the cyclopentane ring fused with imidazole and 4-Cl substituent of (3-(4-chlorophenyl)-1-(2-isopropyl-5-methyl-cyclohexyloxycarbonylmethyl)-6,7-dihydro-5*H*-pyrrolo[1,2-*a*]imidazol-1-ium chloride (**5**) by cycloheptane ring (**12**) and 4-OMe group decreased the activity two fold (MIC = 16 μg/mL/16.8 μM). The presence of 3,4-di-Cl_2_ substitution (compound **11**) appeared to be detrimental, with MIC 32 μg/mL (74.4 μM). In the case of 1,4-diaryl-5,6,7,8-tetrahydro-2a,4a-diazacyclopenta[*cd*]azulenes **13** and **14,** the presence of 4-Me substituent as R’ was beneficial, (MIC = 4 μg/mL/12.3 μM) while introduction of 3,4-di-Cl_2_ substituent as R and replacement of Me by OMe as R’ decreased the activity four times. It is interesting to note the effect of 3,4-Cl_2_ substitution on antibacterial activity. In the case of 3-aryl-1-R-6,7-dihydro-5*H*-pyrrolo[1,2-*a*]imidazol-1-ium chlorides its presence is beneficial, while for 5*H*-imidazo[1,2-*a*]azepine derivatives (**10f**, **11**, **14)** they play negative role.

The compounds displayed only a minor activity against Gram-negative bacteria, with an inhibitory effect only observed for two compounds, namely **6c** towards E. coli, Klebsiella pneumoniae, and Acinetobacter baumannii, and **6d** against A. baumannii only, with MIC values up to 8 μg/mL (15.5 μM). No activity was detected against Pseudomonas aeruginosa for any of studied compounds.

The evaluation of antifungal activity showed no, or only weak, activity for the compounds for inhibiting the growth of C. albicans or C. neoformans. A few compounds (**6a**–**c**, **12** and **14**) displayed MIC values of 32 μg/mL against C. albicans. On the other hand nine compounds displayed MIC values against C. neoformans in the range of 8–16 μg/mL (15.5–34.8 μM). The greatest activity was thereby shown by compound **6c** (MIC is 8 μg/mL/15.5 μM), being equipotent with fluconazole, while compounds **6e**, **10c** and **12** appeared to be two times less potent than **6c**.

According to the results of cytotoxicity testing, most compounds displayed a significant level of cytotoxicity against HEK-293 (human embryonic kidney) cells, with most CC_50_ values similar to the MIC values or only slightly above.

Among the studied *5H*-pyrrolo[1,2-a]imidazole and *5H*-imidazo[1,2-a]azepine quaternary salts, compound **6c** is of special interest. This derivative had a significant activity (MIC up to 4 μg/mL/7.8 μM), as well as a broad spectrum of action, against most of the tested microorganisms (except C. albicans and P. aeruginosa), unfortunately it also displays a similar activity against mammalian cells, as well as hemolytic activity against human red blood cells, indicating that the antibacterial effect is probably due to cytotoxicity. It is interesting to note that 1,4-diaryl-5,6,7,8-tetrahydro-2a,4a-diazacyclopenta[cd]azulenes **13** and **14** had a low level of hemolytic activity against human red blood cells, so heterocyclization of phenacyl *5H*-pyrrolo[1,2-a]imidazole and *5H*-imidazo[1,2-a]azepine quaternary salts to the appropriate diazacyclopenta[cd]azulenes may be one option for solving the problem of the cytotoxicity of the studied class of heterocycles.

For a more detailed biological evaluation of active compounds **6c**, **12**, and **14**, their in vivo acute toxicity was studied in mice (Table 2). In general, the studied compounds possessed satisfactory in vivo toxicity. Thus, the clinical picture of acute intoxication by compound **6c** included decreased motor activity, gait disturbance, lethargy, and tremor in animals receiving doses ≥1000 mg/kg. The death of animals was not recorded. The obtained data made it possible to classify compounds **12** and **14** as III toxicity class, while compound **6c** was IV toxicity class [27].

## 3. Materials and Methods

### 3.1. Chemistry

#### 3.1.1. General Information

All solvents were purified before use. Ethyl acetates were purchased from Acros Organics (Geel, Belgium) and used without purification. Reactions were monitored by thin-layer chromatography (TLC) using Fluka silica gel (60 F 254) plates (0.25 mm). Visualization was performed with UV light. The melting points of the synthesized compounds were taken on a melting point tube. The ^1^H NMR spectra were recorded on a Varian Gemini 400 MHz and ^13^C NMR spectra on a Varian Mercury-400 100 MHz in DMSO-*d_6_* using tetramethylsilane as an internal standard. Chemical shifts are reported in ppm units with use of the d scale. The mass spectra were recorded on an Agilent 1200 LC/MSD SL instrument (Santa Clara, CA, USA).

#### 3.1.2. Synthesis of 3-Aryl-6,7-dihydro-5*H*-pyrrolo[1,2-*a*]imidazole Quaternary Salts (**4**, **5**, **6a**–**e**)

A mixture of 3-aryl-6,7-dihydro-*5H*-pyrrolo[1,2-*a*]imidazoles (**3a**–**e**) (0.01 mol) and appropriate alkylating reagent (0.01 mol) was refluxed for 2 h in 80 mL of ethyl acetate and left overnight at room temperature. The obtained solid products were collected by filtration, washed with ethyl acetate, and recrystallized from the appropriate solvent.

1-Benzyl-3-phenyl-6,7-dihydro-5*H*-pyrrolo[1,2-*a*]imidazol-1-ium chloride (**4**) was obtained using the method from [17].

*3-(4-Chlorophenyl)-1-(2-isopropyl-5-methyl-cyclohexyloxycarbonylmethyl)-6,7-dihydro-5H-pyrrolo[1,2-a]imidazol-1-ium chloride (***5***)*, Yield 67%, mp 238–239 °C (*i*-PrOH). ^1^H NMR (400 MHz, DMSO-*d_6_*) δ: 0.74 (d, 3H, CH_3_, *J* = 6.9 Hz), 0.89 (m, 7H, CH(CH_3_)_2_ + CH), 1.3 and 1.09 (d-d, 2H, CH_2_, *J* = 11.7 Hz), 1.38–2.00 (m, 6H, menthyl H), 2.74 (m, 2H, 6-CH_2_), 3.21 (m, 2H, 7-CH_2_), 4.53 (t, 2H, 5-CH_2_, *J* = 7.3 Hz), 4.72 (m, 1H, CH(CH_3_)_2_), 5.26 and 5.36 (d-d, 2H, N^+^CH_2_COO, *J* = 17.9 Hz), 7.64 and 7.71 (d-d, 4H, C_6_H_4_, *J* = 8.8 Hz), 8.10 (s, 1H, 2-CH). ^13^C NMR (100 MHz, DMSO-*d_6_*) δ: 16.2, 20.6, 21.8, 22.9, 23.1, 25.6, 25.7, 30.8, 33.5, 43.3, 48.8, 49.1, 75.9, 123.7, 124.9, 128.2, 129.1, 129.5, 134.3, 154.6, 166.1. MS *m*/*z*: 416.2 [M^+^]. Anal. calcd for C_24_H_32_Cl_2_N_2_O_2_, % Cl 15.7; N 6.20. Found, % Cl 15.6; N 6.27.

*3-(4-Fluorohenyl)-1-[(4-phenoxyphenylcarbamoyl)-methyl]-6,7-dihydro-5H-pyrrolo[1,2-a]imidazol-1-ium chloride (***6a***)*, Yield 71%, mp 194–195 °C *(i*-PrOH). ^1^H NMR (400 MHz, DMSO-*d_6_*) δ: 2.75 (m, 2H, 6-CH_2_), 3.26 (t, 2H, 7-CH_2_, *J* = 7.6 Hz), 4.53 (t, 2H, 5-CH_2_, *J* = 7.6 Hz), 5.24 (s, 2H, N^+^CH_2_CO), 6.96–7.77 (m, 13H, arom. H), 8.09 (s, 1H, 2-H), 11.2 (s, 1H, NH). ^13^C NMR (100 MHz, DMSO-*d_6_*) δ: 23.2, 25.6, 48.5, 50.8, 116.4, 116.6, 118.0, 119.4, 121.0, 122.68, 122.71, 123.1, 123.3, 128.9, 129.0, 129.1, 130.0, 134.3, 152.2, 154.5, 157.1, 161.4, 163.1, 163.8. MS *m*/*z*: 428.2 [M^+^]. Anal. calcd for C_26_H_23_ClFN_3_O_2_, % Cl 7.65; N 9.05. Found, % Cl 7.69; N 9.21.

*3-(4-Chlorohenyl)-1-[(4-phenoxyphenylcarbamoyl)-methyl]-6,7-dihydro-5H-pyrrolo[1,2-a]imidazol-1-ium chloride* (**6b**), Yield 77%, mp 156–157 °C (*i*-PrOH). ^1^H NMR (400 MHz, DMSO-*d_6_*) δ: 2.75 (m, 2H, 6-CH_2_), 3.26 (t, 2H, 7-CH_2_, *J* = 7.6 Hz), 4.54 (t, 2H, 5-CH_2_, *J* = 7.6 Hz), 5.24 (s, 2H, N^+^CH_2_CO), 6.96–7.74 (m, 13H, arom. H), 8.15 (s, 1H, 2-H), 11.20 (s, 1H, NH). ^13^C NMR (100 MHz, DMSO-*d_6_*) δ: 23.2, 25.6, 48.7, 50.8, 118.0, 119.4, 121.0, 123.1, 123.8, 125.0, 128.1, 129.0, 129.4, 129.9, 134.2, 134.3, 152.2, 154.8, 157.1, 163.1. MS *m*/*z*: 444.2 [M^+^]. Anal. calcd for C_26_H_23_Cl_2_N_3_O_2_, % Cl 14.8; N 8.74. Found, % Cl 15.0; N 8.87.

*3-(3,4-Dichlorohenyl)-1-[(4-phenoxyphenylcarbamoyl)-methyl]-6,7-dihydro-5H-pyrrolo[1,2-a]imidazol-1-ium chloride* (**6c**), Yield 80%, mp 218–219 °C (*i*-PrOH). ^1^H NMR (400 MHz, DMSO-*d_6_*) δ: 2.75 (m, 2H, 6-CH_2_), 3.27 (t, 2H, 7-CH_2_, *J* = 7.6 Hz), 4.57 (t, 2H, 5-CH_2_, *J* = 7.6 Hz), 5.26 (s, 2H, N^+^CH_2_CO), 6.96–8.01 (m, 13H, arom. H), 8.24 (s, 1H, 2-H), 11.2 (s, 1H, NH). ^13^C NMR (100 MHz, DMSO-*d_6_*) δ: 23.2, 25.5, 25.6, 48.7, 50.9, 118.0, 119.4, 121.0, 123.1, 124.6, 126.5, 126.7, 127.8, 128.2, 129.9, 131.5, 132.2, 134.2, 152.3, 155.1, 157.1, 163.0. MS *m*/*z*: 479.3 [M^+^]. Anal. calcd for C_26_H_22_Cl_3_N_3_O_2_, % Cl 20.7; N 8.16. Found, % Cl 20.6; N 8.07.

*3-(4-Methoxyphenyl)-1-[(4-phenoxyphenylcarbamoyl)-methyl]-6,7-dihydro-5H-pyrrolo[1,2-a]imidazol-1-ium chloride* (**6d**), Yield 69%, mp 215–216 °C (i-PrOH). ^1^H NMR (400 MHz, DMSO-d_6_) δ: 2.75 (m, 2H, 6-CH_2_), 3.24 (t, 2H, 7-CH_2_, *J* = 7.3 Hz), 3.83 (s, 3H, OCH_3_), 4.50 (t, 2H, 5-CH_2_, *J* = 7.3 Hz), 5.22 (s, 2H, N^+^CH_2_CO), 6.96–7.69 (m, 13H, arom. H), 7.98 (s, 1H, 2-H), 11.10 (s, 1H, NH). ^13^C NMR (100 MHz, DMSO-*d_6_*) δ: 23.1, 25.6, 48.4, 50.7, 55.4, 114.8, 117.9, 118.0, 118.4, 119.4, 121.0, 122.2, 128.0, 129.9, 130.0, 134.3, 152.1, 154.0, 157.2, 160.1, 163.2. MS *m*/*z*: 440.2 [M^+^]. Anal. calcd for C_27_H_26_ClN_3_O_3_, % Cl 7.46; N 8.82. Found, % Cl 7.60; N 8.71.

*1-[(4-Phenoxyphenylcarbamoyl)-methyl]-3-p-tolyl-6,7-dihydro-5H-pyrrolo[1,2-a]imidazol-1-ium chloride* (**6e**), Yield 72%, mp 248–249 °C (i-PrOH). ^1^H NMR (400 MHz, DMSO-*d_6_*) δ: 2.38 (s, 3H, CH_3_), 2.75 (m, 2H, 6-CH_2_), 3.25 (t, 2H, 7-CH_2_, *J* = 7.4 Hz), 4.52 (t, 2H, 5-CH_2_, *J* = 7.4 Hz), 5.23 (s, 2H, N^+^CH_2_CO), 6.96–7.69 (m, 13H, arom. H), 8.05 (s, 1H, 2-H), 11.1 (s, 1H, NH). ^13^C NMR (100 MHz, DMSO-d_6_) δ: 20.8, 23.1, 25.6, 48.5, 50.7, 118.0, 119.4, 121.0, 122.8, 123.1, 123.3, 126.2, 129.9, 130.0, 130.1, 134.3, 139.3, 152.2, 154.3, 157.1, 163.2. MS *m*/*z*: 425.2 [M^+^]. Anal. calcd for C_27_H_26_ClN_3_O_2_, % Cl 7.72; N 9.13. Found, % Cl 7.61; N 9.25.

#### 3.1.3. Synthesis of 3-Aryl-6,7,8,9-tetrahydro-5*H*-imidazo[1,2-*a*]azepines Quaternary Salts (**9**–**12**)

A mixture of 3-aryl-6,7,8,9-tetrahydro-5*H*-imidazo[1,2-*a*]azepines **8a**–**d** (0.01 mol) and appropriate alkylating reagent (0.01 mol) was refluxed for 2 h in 80 mL of ethyl acetate and left overnight at room temperature. The obtained solid products were collected by filtration, washed with ethyl acetate, and recrystallized from the appropriate solvent.

*1-(3,4-Dichlorobenzyl)-3-phenyl-6,7,8,9-tetrahydro-5H-imidazo[1,2-a]azepin-1-ium bromide* (**9**), Yield 59%, mp 196–197 °C (i-PrOH). ^1^H NMR (400 MHz, DMSO-*d_6_*) δ: 1.70 (m, 2H, 8-CH_2_), 1.87 (m, 4H, 6,7-CH_2_CH_2_), 3.25 (m, 2H, 9-CH_2_), 4.18 (m, 2H, 5-CH_2_), 6.62 (s, 2H, N^+^CH_2_), 7.77 (s, 1H, 2-CH), 7.33–7.79 (m, 8H, C_6_H_5_ + C_6_H_3_). ^13^C NMR (100 MHz, DMSO-*d_6_*) δ: 23.0, 24.0, 25.9, 28.9, 47.3, 48.5, 119.2, 125.5, 128.0, 128.8, 129.2, 129.3, 129.8, 130.1, 131.3, 133.2, 133.8, 134.1, 150.0. MS *m*/*z*: 373.2 [M^+^]. Anal. calcd for C_21_H_21_BrCl_2_N_2_, % N 6.19. Found, % N 6.29.

*1-[2-(2-Difluoromethoxyphenyl)-2-oxoethyl]-3-phenyl-6,7,8,9-tetrahydro-5H-imidazo[1,2-a]azepin-1-ium bromide* (**10a**), Yield 67%, mp 194–194 °C (i-PrOH). ^1^H NMR (400 MHz, DMSO-*d_6_*) δ: 1.73 (m, 2H, 8-CH_2_), 1.88 (m, 4H, 6,7-CH_2_CH_2_), 3.16 (m, 2H, 9-CH_2_), 4.22 (m, 2H, 5-CH_2_), 5.95 (s, 2H, N^+^CH_2_CO), 7.43 (t, 1H, OCHF_2_, *J* = 72.9 Hz), 7.71 (s, 1H, 2-CH), 7.40–8.05 (m, 9H, C_6_H_5_ + C_6_H_4_). ^13^C NMR (100 MHz, DMSO-*d_6_*) δ: 23.1, 23.9, 25.5, 26.3, 28.9, 47.1, 57.3, 113.9, 116.4 t (*J* = 259.2 Hz), 119.0, 118.8, 119.8, 125.4, 125.5, 125.9, 129.3, 129.7, 130.1, 130.8, 133.0, 135.6, 150.4, 151.0, 190.4. MS *m*/*z*: 398.2 [M^+^]. Anal. calcd for C_23_H_23_BrF_2_N_2_O_2_, % N 5.87. Found, % N 5.94.

*1-[2-(4-Isopropylphenyl)-2-oxoethyl]-3-phenyl-6,7,8,9-tetrahydro-5H-imidazo[1,2-a]azepin-1-ium bromide* (**10b**), Yield 61%, mp 225–226 °C (i-PrOH). ^1^H NMR (400 MHz, DMSO-*d_6_*) δ: 1.26 (d, 6H, CH(CH_3_)_2_, *J* = 7.1 Hz), 1.72 (m, 2H, 8-CH_2_), 1.88 (m, 4H, 6,7-CH_2_CH_2_), 3.03 (m, 1H, CH(CH_3_)_2_), 3.16 (m, 2H, 9-CH_2_), 4.23 (m, 2H, 5-CH_2_), 6.15 (s, 2H, N^+^CH_2_CO), 7.69 (s, 1H, 2-CH), 7.49–8.03 (m, 9H, C_6_H_5_ + C_6_H_3_). ^13^C NMR (100 MHz, DMSO-*d_6_*) δ: 23.2, 23.4, 23.9, 26.3, 28.9, 33.6, 47.1, 54.7, 119.9, 125.5, 126.9, 128.7, 129.3, 129.7, 130.1, 131.6, 133.1, 150.9, 155.8, 190.8. MS *m*/*z*: 373.4 [M^+^]. Anal. calcd for C_25_H_29_BrN_2_O, % Br 17.6; N 6.18. Found, % Br 17.7; N 6.25.

*1-[2-Oxo-2-(4-phenoxyphenyl)-ethyl]-3-phenyl-6,7,8,9-tetrahydro-5H-imidazo[1,2-a]azepin-1-ium bromide* (**10c**), Yield 79%, mp 240–241 °C (i-PrOH). ^1^H NMR (400 MHz, DMSO-*d_6_*) δ: 1.72 (m, 2H, 8-CH_2_), 1.88 (m, 4H, 6,7-CH_2_CH_2_), 3.16 (m, 2H, 9-CH_2_), 4.23 (m, 2H, 5-CH_2_), 6.15 (s, 2H, N^+^CH_2_CO), 7.70 (s, 1H, 2-CH), 7.15–8.13 (m, 14H, arom. H). ^13^C NMR (100 MHz, DMSO-*d_6_*) δ: 23.2, 23.9, 25.5, 26.3, 28.9, 47.1, 54.5, 117.4, 119.9, 120.0, 125.0, 125.5, 128.4, 129.3, 129.7, 130.1, 131.1, 133.1, 151.0, 154.8, 162.3, 189.8. MS *m*/*z*: 424.2 [M^+^]. Anal. calcd for C_28_H_27_BrN_2_O_2_, % Br 15.9; N 5.56. Found, % Br 15.7; N 5.64.

*1-[2-(4-Cyclohexylphenyl)-2-oxoethyl]-3-(4-fluorophenyl)-6,7,8,9-tetrahydro-5H-imidazo[1,2-a]azepin-1-ium bromide* (**10d**), Yield 58%, mp 150–151 °C (i-PrOH). ^1^H NMR (400 MHz, DMSO-*d_6_*) δ: 1.25–1.53 (m, 5H, cyclohexyl H), 1.71–1.90 (m, 11H, 6,7,8-(CH_2_)_3_ + cyclohexyl H), 2.65 (m, 1H, CH), 3.15 (m, 2H, 9-CH_2_), 4.19 (m, 2H, 5-CH_2_), 6.14 (s, 2H, N^+^CH_2_CO), 7.68 (s, 1H, 2-CH), 7.44–8.02 (m, 8H, C_6_H_4_ + C_6_H_3_). ^13^C NMR (100 MHz, DMSO-*d_6_*) δ: 23.2, 23.9, 25.4, 26.1, 26.2, 28.9, 33.5, 43.9, 47.0, 54.6, 115.9, 116.1, 115.9, 116.1, 116.3, 116.5, 120.0, 121.9, 127.3, 128.7, 128.8, 128.9, 131.5, 132.1, 132.3, 132.4, 140.9, 150.9, 154.8, 161.8, 164.3, 190.7. MS *m*/*z*: 431.4 [M^+^]. Anal. calcd for C_28_H_32_BrFN_2_O, % N 5.47. Found, % N 5.33.

*3-(4-Chlorophenyl)-1-[2-(4-cyclohexylphenyl)-2-oxoethyl]-6,7,8,9-tetrahydro-5H-imidazo[1,2-a]azepin-1-ium bromide* (**10e**), Yield 63%, mp 253–254°C (i-PrOH). ^1^H NMR (400 MHz, DMSO-*d_6_*) δ: 1.25–1.51 (m, 5H, cyclohexyl H), 1.71–1.89 (m, 11H, 6,7,8-(CH_2_)_3_ + cyclohexyl H), 2.65 (m, 1H, CH), 3.14 (m, 2H, 9-CH_2_), 4.21 (m, 2H, 5-CH_2_), 6.15 (s, 2H, N^+^CH_2_CO), 7.71 (s, 1H, 2-CH), 7.51 and 7.69 (d-d, 4H, C_6_H_4_, *J* = 8.3 Hz), 7.54 and 8.01 (d-d, 4H, C_6_H_4_, J = 8.3 Hz). ^13^C NMR (100 MHz, DMSO-*d_6_*) δ: 23.1, 23.9, 25.4, 26.1, 26.2, 28.9, 33.5, 43.9, 47.1, 54.6, 120.2, 124.4, 127.3, 128.7, 129.4, 131.5, 131.6, 131.9, 135.1, 151.2, 154.8, 190.7. MS *m*/*z*: 448.4 [M^+^]. Anal. calcd for C_28_H_32_BrClN_2_O, % N 5.30. Found, % N 5.42.

*1-[2-(4-Bromophenyl)-2-oxoethyl]-3-(3,4-dichlorophenyl)-6,7,8,9-tetrahydro-5H-imidazo[1,2-a]azepin-1-ium bromide* (**10f**), Yield 85%, mp 243–244 °C (i-PrOH). ^1^H NMR (400 MHz, DMSO-*d_6_*) δ: 1.71 (m, 2H, 8-CH_2_), 1.87 (m, 4H, 6,7-CH_2_CH_2_), 3.16 (m, 2H, 9-CH_2_), 4.22 (m, 2H, 5-CH_2_), 6.17 (s, 2H, N^+^CH_2_CO), 7.75 (s, 1H, 2-CH), 7.50–7.89 (m, 3H, C_6_H_3_), 7.89 and 8.02 (d-d, 4H, C_6_H_4_). ^13^C NMR (100 MHz, DMSO-*d_6_*) δ: 23.1, 23.9, 25.5, 26.1, 28.8, 47.4, 54.8, 120.8, 126.1, 128.7, 130.1, 130.4, 130.8, 131.4, 131.8, 132.1, 132.7, 133.2, 151.3, 190.6. MS *m*/*z*: 479.0 [M^+^]. Anal. calcd for C_22_H_20_Br_2_Cl_2_N_2_O, % N 5.01. Found, % N 5.13.

3-Phenyl-1-[2-(4-methylphenyl)-2-oxoethyl]-6,7,8,9-tetrahydro-5*H*-imidazo[1,2-*a*]azepin-1-ium bromide (**10g**) was obtained using the method of [19].

*3-(3,4-Dichlorophenyl)-1-[2-(4-methoxyphenyl)-2-oxoethyl]-6,7,8,9-tetrahydro-5H-imidazo[1,2-a]azepin-1-ium bromide* (**10h**), Yield 80%, mp 172–173 °C (i-PrOH). ^1^H NMR (400 MHz, DMSO-*d_6_*) δ: 1.72 (m, 2H, 8-CH_2_), 1.84 (m, 4H, 6,7-CH_2_CH_2_), 3.14 (m, 2H, 9-CH_2_), 4.23 (m, 2H, 5-CH_2_), 6.18 (s, 2H, N+CH_2_CO), 7.17 and 8.07 (d-d, 4H, C_6_H_4_, *J* = 8.8 Hz), 7.81 (s, 1H, 2-CH), 7.51–7.90 (m, 3H, C_6_H_3_). MS *m*/*z*: 429.0 [M^+^]. Anal. Calcd for C_23_H_23_BrCl_2_N_2_O_2_, % N 5.49. Found, % N 5.57.

*3-(3,4-Dichlorophenyl)-1-[(isopropylphenylcarbamoyl)-methyl]-6,7,8,9-tetrahydro-5H-imidazo[1,2-a]azepin-1-ium chloride* (**11**), Yield 77%, mp 164–165 °C (i-PrOH). ^1^H NMR (400 MHz, DMSO-*d_6_*) δ: 1.05 (d, 6H, CH(CH_3_)_2_, *J* = 6.6 Hz), 1.75 (m, 2H, 8-CH2), 1.80 (m, 2H, 7-CH_2_), 1.88 (m, 2H, 6-CH_2_), 2.54 (d, 3H, CH_3_, *J* = 6.4 Hz), 3.08 (m, 2H, 9-CH_2_), 4.18 (m, 2H, 5-CH_2_), 4.75 (m, 1H, CH(CH_3_)_2_), 4.76 (s, 2H, N^+^CH_2_CO), 7.77 (s, 1H, 2-CH), 7.46–7.87 (m, 3H, C_6_H_5_ + C_6_H_3_). ^13^C NMR (100 MHz, DMSO-*d_6_*) δ: 20.4, 23.0, 24.3, 26.1, 28.7, 47.0, 47.3, 50.3, 121.1, 126.1, 129.1, 129.6, 129.9, 130.2, 130.4, 131.4, 131.5, 135.5, 132.0, 133.0, 135.9, 151.3, 163.4. MS *m*/*z*: 458.2 [M^+^]. Anal. calcd for C_25_H_28_Cl_3_N_3_O, % Cl 21.6; N 8.52. Found, % Cl 21.8; N 8.66.

*1-(2-Isopropyl-5-methyl-cyclohexyloxycarbonylmethyl)-3-(4-methoxyphenyl)-6,7,8,9-tetrahydro-5H-imidazo[1,2-a]azepin-1-ium chloride* (**12**), Yield 70%, mp 139–140 °C (i-PrOH). ^1^H NMR (400 MHz, DMSO-*d_6_*) δ: 0.72 (d, 3H, CH_3_, *J* = 6.8 Hz), 0.89 (m, 7H, CH(CH_3_)_2_ + CH), 1.01–1.98 (m, 14H, 6,7,8-(CH_2_)_3_ + menthyl H), 3.16 (m, 2H, 9-CH_2_), 3.84 (s, 3H, OCH_3_), 4.16 (m, 2H, 5-CH_2_), 4.71 (m, 1H, CH(CH_3_)_2_), 5.37 and 5.49 (d-d, 2H, N^+^*CH*_2_COO, *J* = 18.1 Hz), 7.15 and 7.39 (d-d, 4H, C_6_H_4_, *J* = 8.8 Hz), 7.74 (s, 1H, 2-C*H*). ^13^C NMR (100 MHz, DMSO-*d_6_*) δ: 16.2, 20.5, 21.8, 22.8, 23.0, 23.9, 25.6, 26.2, 28.8, 30.8, 33.5, 46.4, 46.8, 48.8, 55.4, 76.0, 114.8, 117.2, 119.5, 131.3, 133.0, 150.2, 160.6, 166.4. MS *m*/*z*: 439.4 [M^+^]. Anal. calcd for C_27_H_39_ClN_2_O_3_, % Cl 7.47; N 5.89. Found, % Cl 7.61; N 5.97.

#### 3.1.4. Synthesis of 1,4-Diaryl-5,6,7,8-tetrahydro-2a,4a-diazacyclopenta[*cd*]azulenes (**13**,**14**)

The appropriate 3-aryl-6,7,8,9-tetrahydro-5*H*-imidazo[1,2-*a*]azepine quaternary salt **10g**,**h** (0.01 mol) was refluxed for 1 h in 80 mL of 5% NaOH in water and left overnight at room temperature. The obtained solid products were collected by filtration, washed with water, and recrystallized from benzene/heptane (1:1).

4-Phenyl-1-p-tolyl-5,6,7,8-tetrahydro-2a,4a-diazacyclopenta[cd]azulene (**13**) was obtained by method [19].

*4-(3,4-Dichlorophenyl)-1-(4-methoxyphenyl)-5,6,7,8-tetrahydro-2a,4a-diazacyclopenta[cd]azulene* (**14**), Yield 89%, mp 158–159 °C. ^1^H NMR (300 MHz, DMSO-*d_6_*) δ: 1.86 (m, 2H, 6-CH_2_), 2.01 (m, 2H, 7-CH_2_), 2.75 (m, 2H, 8-CH_2_), 3.70 (m, 2H, 5-CH_2_), 3.76 (s, 3H, OCH_3_), 6.80 (s, 1H, 2-CH), 6.92 and 7.34 (d-d, 4H, C_6_H_4_, *J* = 8.4 Hz), 7.39 (s, 1H, 3-CH), 7.48 and 7.69 (d-d, 2H, C_6_H_3_, *J* = 8.6 Hz), 7.75 (s, 1H, C_6_H_3_). MS *m*/*z*: 410.0 [M^+^]. Anal. calcd for C_23_H_20_Cl_2_N_2_O, % Cl 17.2; N 6.81. Found, % Cl 17.4; N 6.93.

### 3.2. Biological Activity

#### 3.2.1. Compound Preparation

The tests were carried out initially at a single compound concentration of 32 µg/mL in duplicates, to identify any actives, and followed up by a hit confirmation of the active compounds by a dose response test, using 8 concentrations at 1:2 dilution, in duplicates, to determine the minimum inhibitory concentration (MIC) against bacteria and yeasts, CC_50_ against mammalian cells and HC_10_ against human red blood cells.

All compounds were dissolved in DMSO to give a stock concentration of 10 mg/mL, and aliquots were diluted in water and 5 µL was dispensed into empty 384-well plates in duplicates for each strain and cell assayed. Once cells were added to the plates, this gave a final compound concentration of 32 µg/mL, or in the case of a serial dilution assay compound, concentrations from 32 to 0.25 µg/mL, in both cases with a maximum DMSO concentration of 0.3%.

#### 3.2.2. Antimicrobial Assays

The compounds were tested for activity against 1 Gram-positive bacteria (Staphylococcus aureus *ATCC* 43,300 MRSA), 4 Gram-negative bacteria (*Escherichia coli ATCC* 25922, *Pseudomonas aeruginosa ATCC* 27853, *Klebsiella pneumoniae* ESBL *ATCC* 700603, Acinetobacter baumannii *ATCC* 19606), and 2 yeasts (*Candida albicans ATCC* 90028, *Cryptococcus neoformans* H99 *ATCC* 208821); and all tests were carried out by the Community for Open Antimicrobial Drug Discovery (CO-ADD).

All bacteria were cultured in Cation-adjusted Mueller Hinton broth (CAMHB) at 37 °C overnight. The resultant mid-log phase cultures were added to each well of the compound containing plates (384-well non-binding surface plates—Corning 3640), giving a cell density of 5 × 10^5^ CFU/mL (colony forming units/mL). All plates were covered and incubated at 37 °C for 18 h without shaking. Inhibition of bacterial growth was determined by measuring absorbance at 600 nm (OD600) using a Tecan M1000 Pro monochromator plate reader.

Yeast strains were cultured for 3 days on yeast extract-peptone dextrose (YPD) agar at 30 °C. A yeast suspension of 1 × 10^6^ to 5 × 10^6^ CFU/mL (as determined by OD_530_) was prepared from five colonies. These stock suspensions were diluted with yeast nitrogen base (YNB) broth to a final concentration of 2.5 × 103 CFU/mL. Then, 45 μL of the yeast suspension was added to each well of the compound containing plates (384-well non-binding surface plates; Corning 3640). Plates were covered and incubated at 35 °C for 24 h without shaking. Growth inhibition of *C. albicans* was determined by measuring the absorbance at 530 nm (OD_530_), while the growth inhibition of *C. neoformans* was determined by measuring the difference in absorbance between 600 and 570 nm (OD_600–570_), after the addition of resazurin (0.001% final concentration) and incubation at 35 °C for an additional 2 h. The absorbance was measured using a Biotek Synergy HTX plate reader.

Growth inhibition was calculated as the percentage difference between untreated cells (positive growth control) and media only (negative growth control). Compounds with ≥80% growth inhibition were selected as actives in the initial screening, and MIC was determined following EUCAST recommendations, using 80% growth inhibition as a threshold for full inhibition.

Colistin sulfate (Sigma Aldrich, St. Louis, MO, USA; Cat# C4461) and vancomycin HCl (Sigma Aldrich, St. Louis, MO, USA; Cat# 861987) were used as positive bacterial inhibitor standards for Gram-negative and Gram-positive bacteria, respectively. Fluconazole (Sigma Aldrich, St. Louis, MO, USA; Cat# F8929) was used as a positive yeast inhibitor standard for *C. albicans* and C. *neoformans*.

#### 3.2.3. Cytotoxicity Assay

*HEK-293* (human embryonic kidney) *ATCC* CRL-1573 cells were counted manually in a Neubauer hemocytometer and then plated in 384-well tissue culture treated plates (Corning 3712) containing the compounds to give a density of 5000 cells/well in a final volume of 50 μL. DMEM supplemented with 10% FBS was used as growth media, and the cells were incubated together with the compounds for 20 h at 37 °C in 5% CO_2_. Cytotoxicity (or cell viability) was measured by fluorescence, ex: 560/10 nm, em: 590/10 nm (F_560/590_), after addition of 5 μL of 25 μg/mL resazurin (2.3 μg/mL final concentration) and after incubation for a further 3 h at 37 °C in 5% CO_2_. The fluorescence intensity was measured using a Tecan M1000 Pro monochromator plate reader, using automatic gain calculation. CC_50_ (concentration at 50% cytotoxicity) was calculated by curve fitting the inhibition values vs. log (concentration) using a sigmoidal dose–response function, with variable fitting values for bottom, top, and slope.

#### 3.2.4. Hemolysis Assay

Human whole blood was washed three times with 3 volumes of 0.9% NaCl and then resuspended in the same to a concentration of 0.5 × 108 cells/mL, as determined by manual cell count in a Neubauer hemocytometer. The washed cells were then added to the 384-well compound containing polystyrene plates (Corning 3657) for a final volume of 50 μL. After a 10 min shake on a plate shaker, the plates were incubated for 1 h at 37 °C. After incubation, the plates were centrifuged at 1000× *g* for 10 min to pellet cells and debris, and 25 μL of the supernatant was then transferred to a polystyrene 384-well assay plate (Corning 3680). Hemolysis was determined by measuring the supernatant absorbance at 405 mm (OD_405_). The absorbance was measured using a Tecan M1000 Pro monochromator plate reader. HC_10_ (concentration causing 10% hemolysis) were calculated by curve fitting the inhibition values vs. log (concentration) using a sigmoidal dose–response function with variable fitting values for top, bottom, and slope. The use of human blood (sourced from the Australian Red Cross Blood Service) for hemolysis assays was approved by The University of Queensland Institutional Human Research Ethics Committee, Approval Number 2014000031.

#### 3.2.5. In Vivo Toxicity Assay

The in vivo acute toxicity of compounds **6c**, **12**, and **14** was studied by the oral route of administration in white non-linear female mice weighing 20 ± 2 g. The animals were kept on a standard diet and received food and drink ad libitum. The studies were carried out in accordance with the application for a preclinical study approved by the Institute of Pharmacology and Toxicology Bioethical Committee (Approval Number 3.06.19/25.12.2019). Compounds were administered to animals once intragastrically in the form of a water–alcohol emulsion, using Tween-80 as an emulsifier. The volume of the substance did not exceed 0.5 mL. The solvent used was ethyl alcohol 95% (5%) and distilled water. Animals were randomized into groups of 5 animals each. The compounds were administered in doses: **6c**—100, 500, 1000, 2000 mg/kg; **12**—100, 250, 500, 750, 1000 mg/kg; and **14**—100, 500, 1000, 1500, 2000 mg/kg. During the experiment, the state of the animals and the timing of death were monitored. The results were recorded in an alternative form (the number of dead animals) 14 days after a single injection. Toxicity was calculated using the Litchfield–Wilcoxon method [28].

## 4. Conclusions

Primary screening of the newly synthesized compounds to assess their antibacterial and antifungal properties showed that derivatives of 3-aryl-5*H*-pyrrolo[1,2-*a*]imidazole and 5*H*-imidazo[1,2-*a*]azepine quaternary salts have a high inhibitory effect. Among the newly synthesized compounds, a potential hit **6c**, with a broad spectrum of action against *S. aureus*, *E. coli*, *K. pneumoniae*, *A. baumannii,* and *C. neoformans*, was identified. Notably, compound **6c** possess a high hemolytic activity against human red blood cells and cytotoxicity against *HEK-293*, but possessed a low in vivo toxicity in mice.

## Figures and Tables

**Table 1:** Antimicrobial activity (MIC), cytotoxicity (CC_50_), and hemolytic activity (HC_10_) of the new 3-aryl-*5H*-pyrrolo[1,2-*a*]imidazole and 5*H*-imidazo[1,2-*a*]azepine quaternary salts.

Compounds	MIC, µg/mL (μM)							CC_50_, µg/mL (μM)	HC_10_, µg/mL (μM)
	*Sa* ^1^	*Ec* ^2^	*Kp* ^3^	*Ab* ^4^	*Pa* ^5^	*Ca* ^6^	*Cn* ^7^	*Hek* ^8^	*RBC* ^9^
**4**	na	na	na	na	na	na	na	n.d.	n.d.
**5**	8.0 (17.7)	>32.0	>32.0	>32.0	>32.0	>32.0	32.0 (70.9)	3.8 (8.4)	>32.0
**6a**	8.0 (17.2)	32.0 (69.0)	>32.0	32.0 (69.0)	>32.0	32.0 (69.0)	16.0 (34.5)	18.1 (39.0)	16.5 (35.6)
**6b**	4.0 (8.3)	32.0 (66.6)	>32.0	32.0 (66.6)	>32.0	32.0 (66.6)	16.0 (33.3)	>32	18.2 (37.9)
**6c**	4.0 (7.8)	16.0 (31.1)	8.0 (15.5)	16.0 (31.1)	>32.0	32.0 (62.1)	8.0 (15.5)	10.8 (21.0)	3.7 (7.2)
**6d**	16.0 (33.6)	>32.0	>32.0	16.0 (33.6)	>32.0	>32.0	32.0 (67.2)	>32	>32.0
**6e**	8.0 (17.4)	>32.0	>32.0	32.0 (69.6)	>32.0	>32.0	16.0 (34.8)	18.4 (40.0)	>32.0
**10b**	32.0 (70.6)	>32.0	>32.0	>32.0	>32.0	>32.0	>32.0	11.6 (25.6)	>32.0
**10c**	4.0 (8.0)	32.0 (63.6)	>32.0	32.0 (63.6)	>32.0	>32.0	16.0 (31.8)	4.8 (9.5)	>32.0
**10d**	4.0 (7.8)	>32.0	>32.0	>32.0	>32.0	>32.0	32.0 (62.6)	5.9 (11.5)	>32.0
**10e**	2.0 (3.8)	>32.0	>32.0	32.0 (60.6)	>32.0	32.0 (60.6)	16.0 (30.3)	3.9 (7.4)	14.6 (27.7)
**10f**	16.0 (28.6)	>32.0	>32.0	>32.0	>32.0	>32.0	32.0 (57.2)	8.4 (15.0)	>32.0
**11**	32.0 (74.4)	>32.0	>32.0	>32.0	>32.0	>32.0	>32.0	>32.0	>32.0
**12**	8.0 (16.8)	>32.0	>32.0	>32.0	>32.0	>32.0	16.0 (33.7)	1.4 (3.0)	>32.0
**13**	4.0 (12.3)	>32.0	>32.0	>32.0	>32.0	>32.0	32.0 (98.0)	>32.0	>32.0
**14**	16.0 (38.9)	>32.0	>32.0	>32.0	>32.0	32.0 (77.8)	>32.0	16.5 (40.1)	>32.0
**Vancomycin**	1 (0.69)								
**Colistin**		0.125 (0.11)	0.25 (0.22)	0.25 (0.22)	0.25 (0.22)				
**Fluconazole**						0.125 (0.41)	8.0 (26.1)		
**Tamoxifen**								9.0 (24.2)	
**Melittin**									2.7 (0.95)

^1^ Sa—Staphylococcus aureus (MRSA) ATCC 43300; ^2^ Ec—Escherichia coli ATCC 25922; ^3^ Kp—Klebsiella pneumoniae ESBL ATCC 700603; ^4^ Ab—Acinetobacter baumannii ATCC 19606; ^5^ Pa—Pseudomonas aeruginosa ATCC 27853; ^6^ Ca—Candida albicans ATCC 90028; ^7^ Cn—Cryptococcus neoformans H99 ATCC 208821; ^8^ Hek—HEK-293, human embryonic kidney cells ATCC CRL-1573; ^9^ RBC—human red blood cells; na—not active in primary screening; n.d.—not determined.

**Table 2:** In vivo acute toxicity of compounds **6c**, **12** and **14**.

Compounds	LD_50_, mg/kg	Range of LD_50_, mg/kg
**6c**	>2000	-
**12**	682.8	568.5–820
**14**	805.9	516.7–1275.0

## Data Availability

Data available in a publicly accessible repository.

## Supplementary Materials

The following are available online. Copies of NMR spectra of compounds **5**, **6a**–**e**, **9**–**12**.

## Sample Availability

Samples of the compounds **5**, **6a**–**e**, **9**–**14** are available from the authors.
