# Supplementary material for: Synthesis, Antibacterial and Antifungal Activity of New 3-Aryl-5H-pyrrolo[1,2-a]imidazole and 5H-Imidazo[1,2-a]azepine Quaternary Salts
Source: Molecules. 2021 Jul 13;26(14):4253. doi: 10.3390/molecules26144253 (PMC8305969; doi:10.3390/molecules26144253)
Supplement: Supplementary file 1 [file molecules-26-04253-s001.zip › molecules-1272970-supplementary.pdf]

SUPPLEMENTARY MATERIALS

Synthesis, antibacterial and antifungal activity of new 3-aryl-5*H*-pyrrolo[1,2-*a*]imidazol and 5*H*-imidazo[1,2-*a*]azepin quaternary salts

Sergii Demchenko [1], Roman Lesyk [2,*], Oleh Yadlovskyi [1], Johannes Zuegg [3], Alysha G. Elliott [3], Iryna Drapak [4], Yuliia Fedchenkova [5], Zinaida Suvorova [1], Anatolii Demchenko [1,5]

[1] Institute of Pharmacology and Toxicology, Antona Tsedika 14, 03057 Kyiv, Ukraine.

[2] Department of Public Health, Dietetics and Lifestyle Disorders, Faculty of Medicine, University of Information Technology and Management in Rzeszow, Sucharskiego 2, 35-225 Rzeszow, Poland

[3] The Community for Open Antimicrobial Drug Discovery (CO-ADD), Centre for Superbug Solutions, Institute for Molecular Bioscience, The University of Queensland, Brisbane 4072, Australia

[4] Department of General, Inorganic, Physical and Colloidal Chemistry, Danylo Halytsky Lviv National Medical University, Pekarska 69, 79010 Lviv-10, Ukraine

[5] Nizhyn Mykola Gogol State University, Nizhyn, Ukraine

[*]corresponding author

dr_r_lesyk@org.lviv.net (R. Lesyk)

tel. +38 0322 75-59-66

fax. +38 0322 75-77-34

Copies of NMR spectra

3-(4-Chlorophenyl)-1-(2-isopropyl-5-methyl-cyclohexyloxycarbonylmethyl)-6,7-dihydro-5H-pyrrolo[1,2-a]imidazol-1-ium chloride ***(5)***

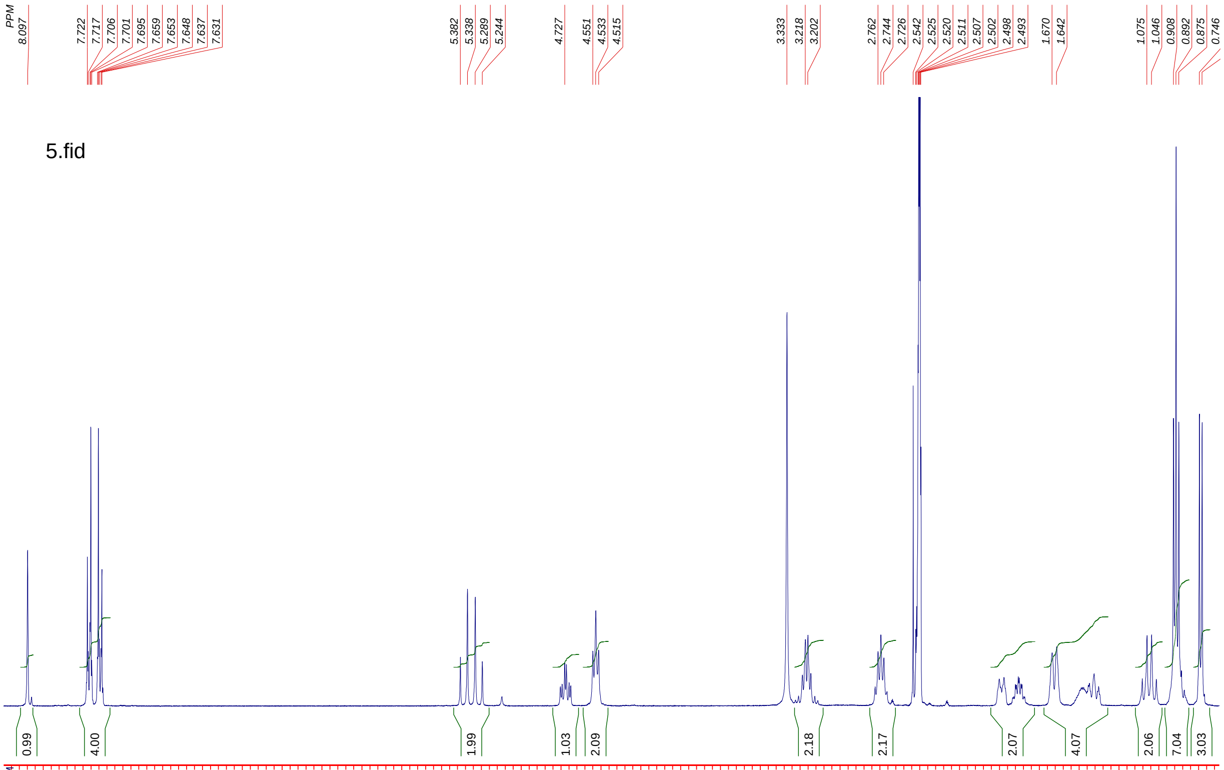

File name: 5.fid	Operator:	SF: 400.0773 MHz	NSC: 0	PW: 5.30 usec, RG: 30	SI: 32768
Date: 13-Jul-2020	Solvent: dmso	SW: 8013 Hz	TE: 293 K	AQ: 2.04 sec, RD: 0.00 sec	dmso new

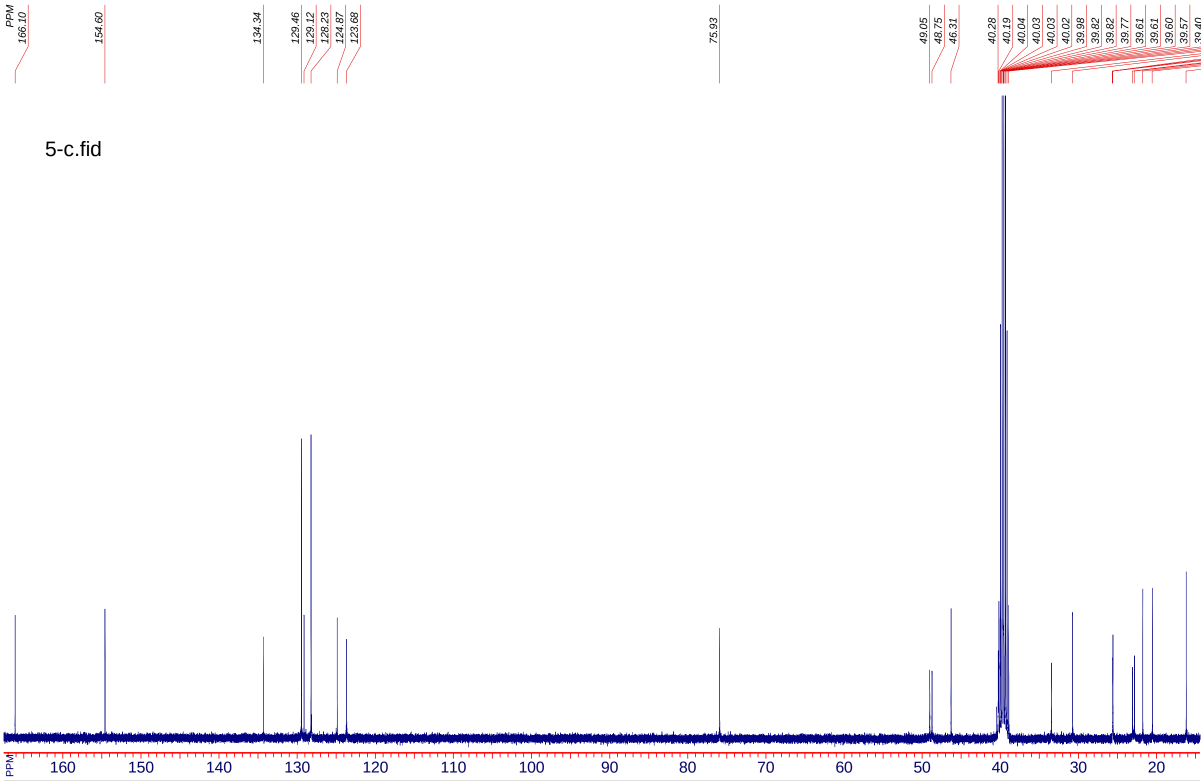

File name: 5-c.fid	Operator:	SF: 100.6117 MHz	NSC: 0	PW: 4.30 usec, RG: 38	SI: 131072
Date: 15-Sep-2020	Solvent: dmso	SW: 28409 Hz	TE: 294 K	AQ: 2.25 sec, RD: 0.00 sec	dmso 0122

*3-(4-Fluorohenyl)-1-[(4-phenoxyphenylcarbamoyl)-methyl]-6,7-dihydro-5H-pyrrolo[1,2-a]imidazol-1-ium chloride (**6a**)*

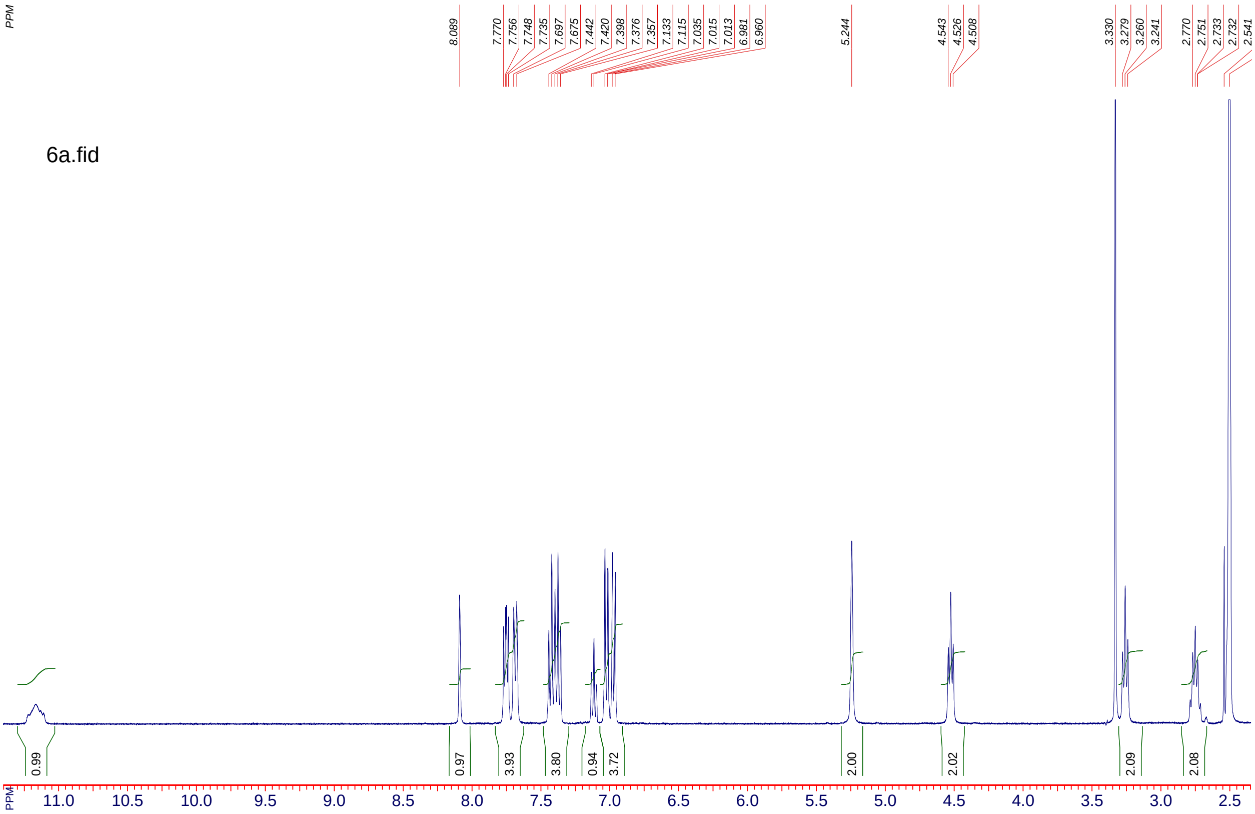

**File name: 6a.fid**	Operator:	SF: 400.0773 MHz	NSC: 0	PW: 5.30 usec, RG: 30	SI: 32768
Date: 13-Jul-2020	Solvent: dmso	SW: 8013 Hz	TE: 293 K	AQ: 2.04 sec, RD: 0.00 sec	dmso new

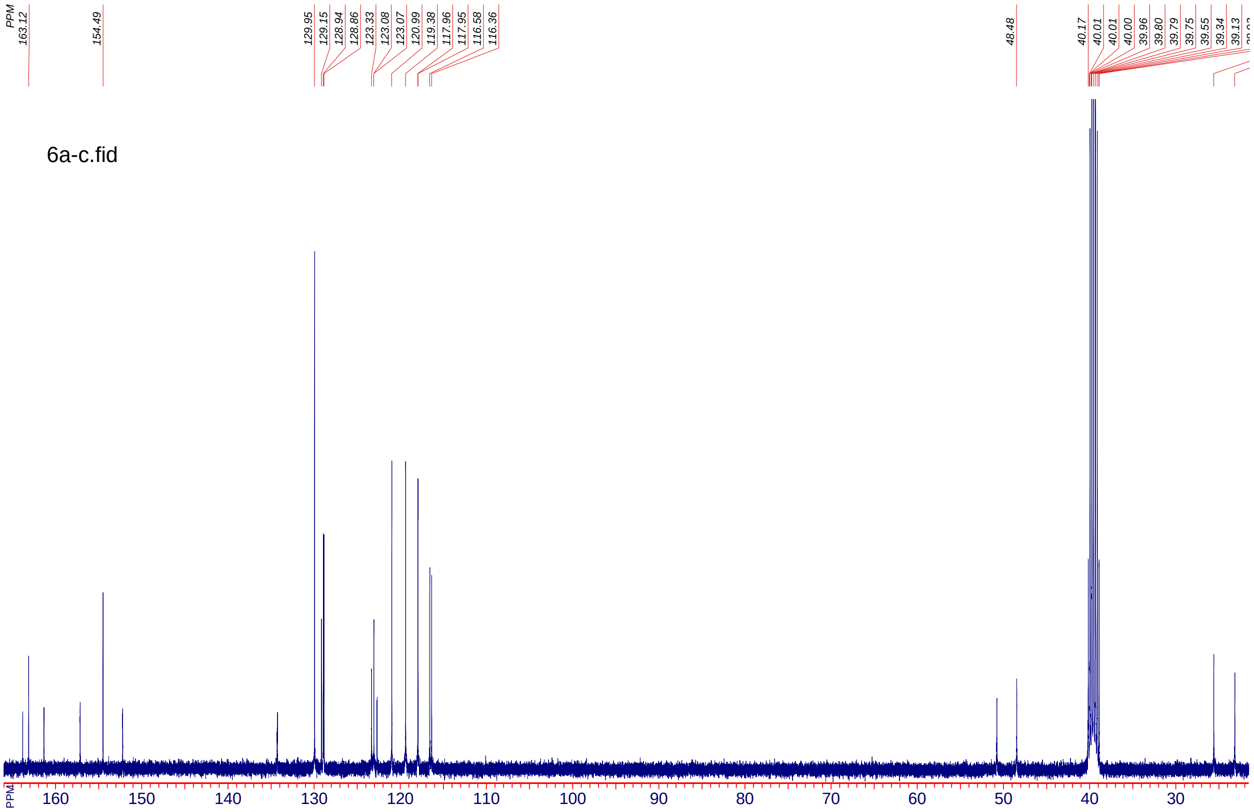

**File name: 6a-c.fid**	Operator:	SF: 100.6117 MHz	NSC: 0	PW: 4.30 usec, RG: 38	SI: 131072
Date: 10-Sep-2020	Solvent: dmso	SW: 28409 Hz	TE: 294 K	AQ: 2.25 sec, RD: 0.00 sec	dmso 0122

*3-(4-Chlorohenyl)-1-[(4-phenoxyphenylcarbamoyl)-methyl]-6,7-dihydro-5H-pyrrolo[1,2-a]imidazol-1-ium chloride (**6b**)*

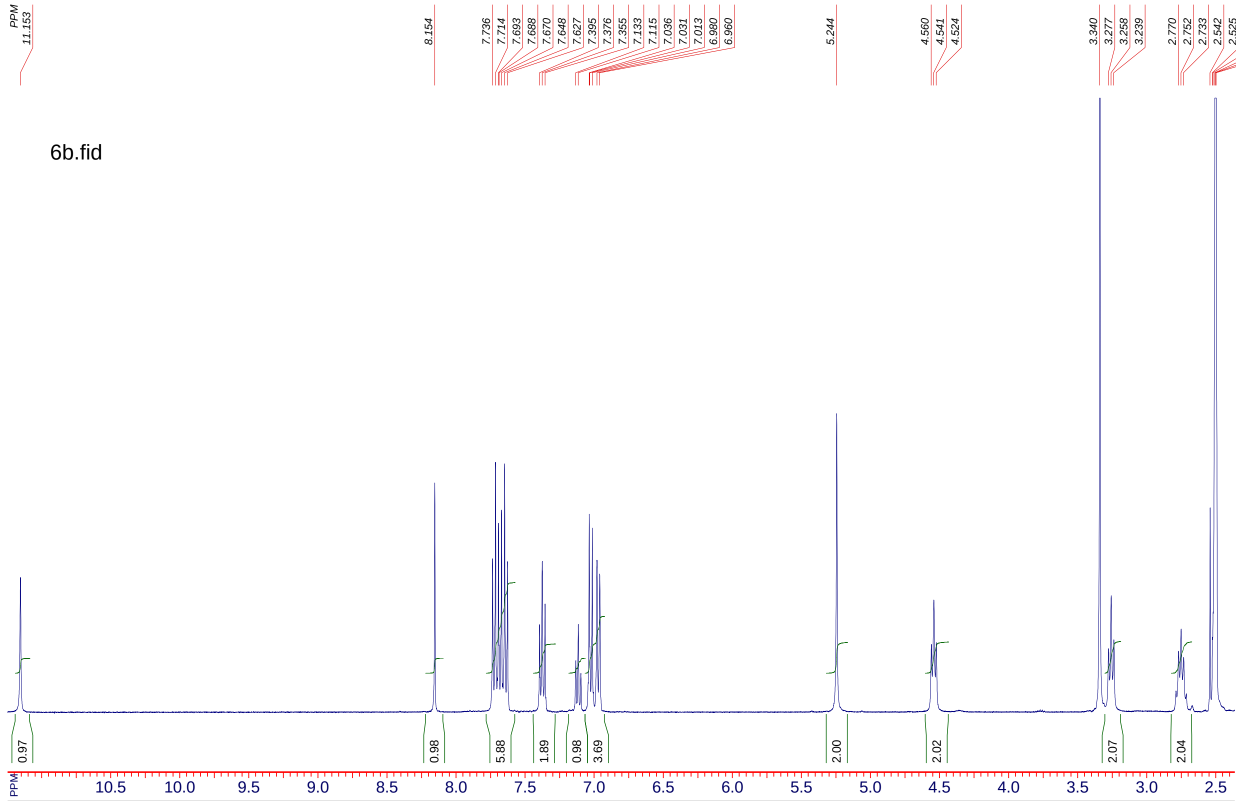

**File name: 6b.fid**	Operator:	SF: 400.0773 MHz	NSC: 0	PW: 5.30 usec, RG: 30	SI: 32768
Date: 13-Jul-2020	Solvent: dmso	SW: 8013 Hz	TE: 293 K	AQ: 2.04 sec, RD: 0.00 sec	dmso new

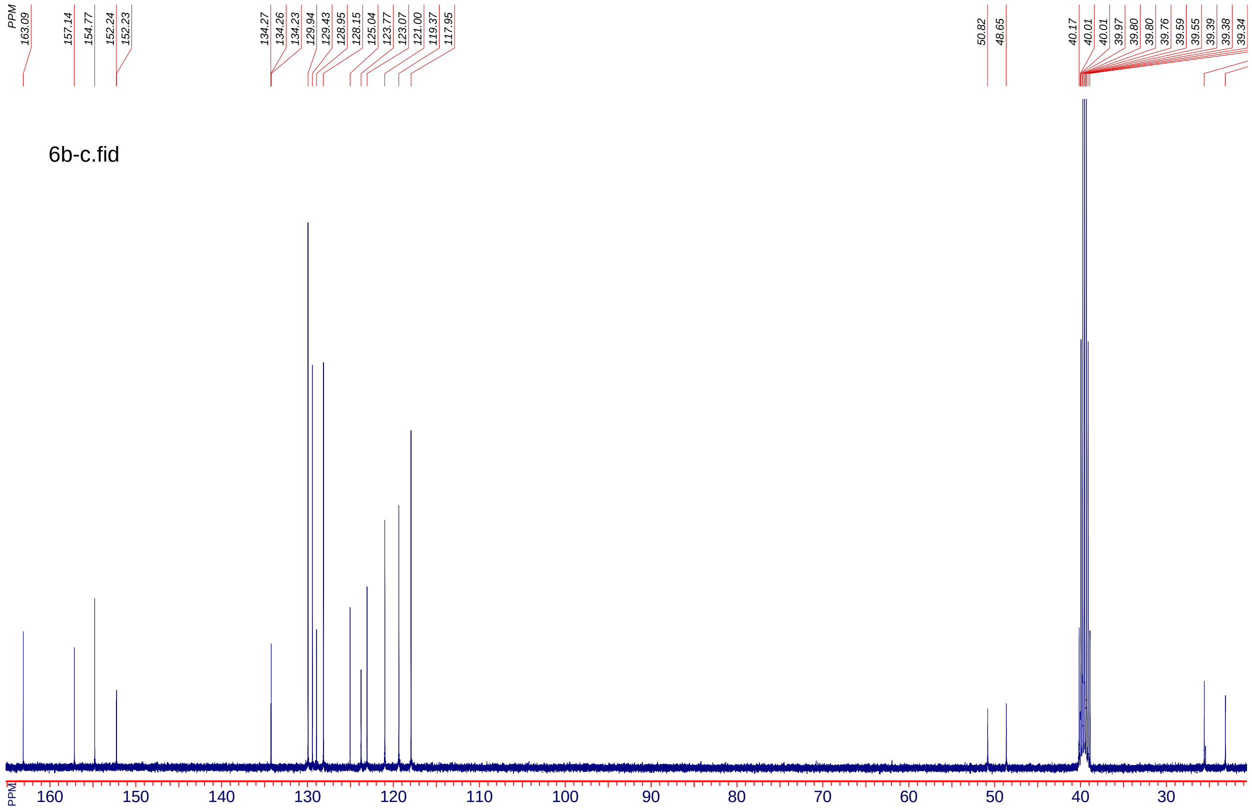

**File name: 6b-c.fid**	Operator:	SF: 100.6117 MHz	NSC: 0	PW: 4.30 usec, RG: 38	SI: 131072
Date: 11-Sep-2020	Solvent: dmso	SW: 28409 Hz	TE: 294 K	AQ: 2.25 sec, RD: 0.00 sec	dmso 0122

*3-(3,4-Dichlorohenyl)-1-[(4-phenoxyphenylcarbamoyl)-methyl]-6,7-dihydro-5H-pyrrolo[1,2-a]imidazol-1-ium chloride (**6c**)*

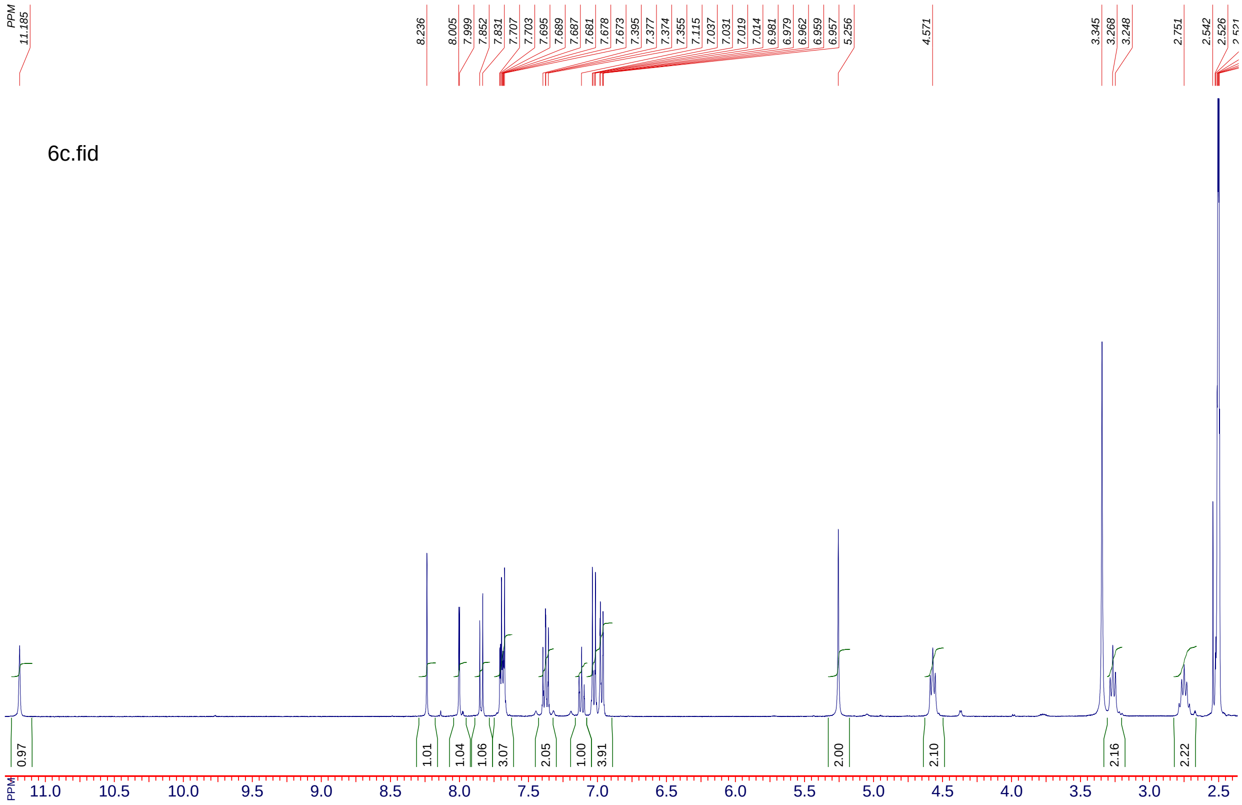

**File name: 6c.fid**	Operator:	SF: 400.0773 MHz	NSC: 0	PW: 5.30 usec, RG: 30	SI: 32768
Date: 13-Jul-2020	Solvent: dmso	SW: 8013 Hz	TE: 293 K	AQ: 2.04 sec, RD: 0.00 sec	dmso new

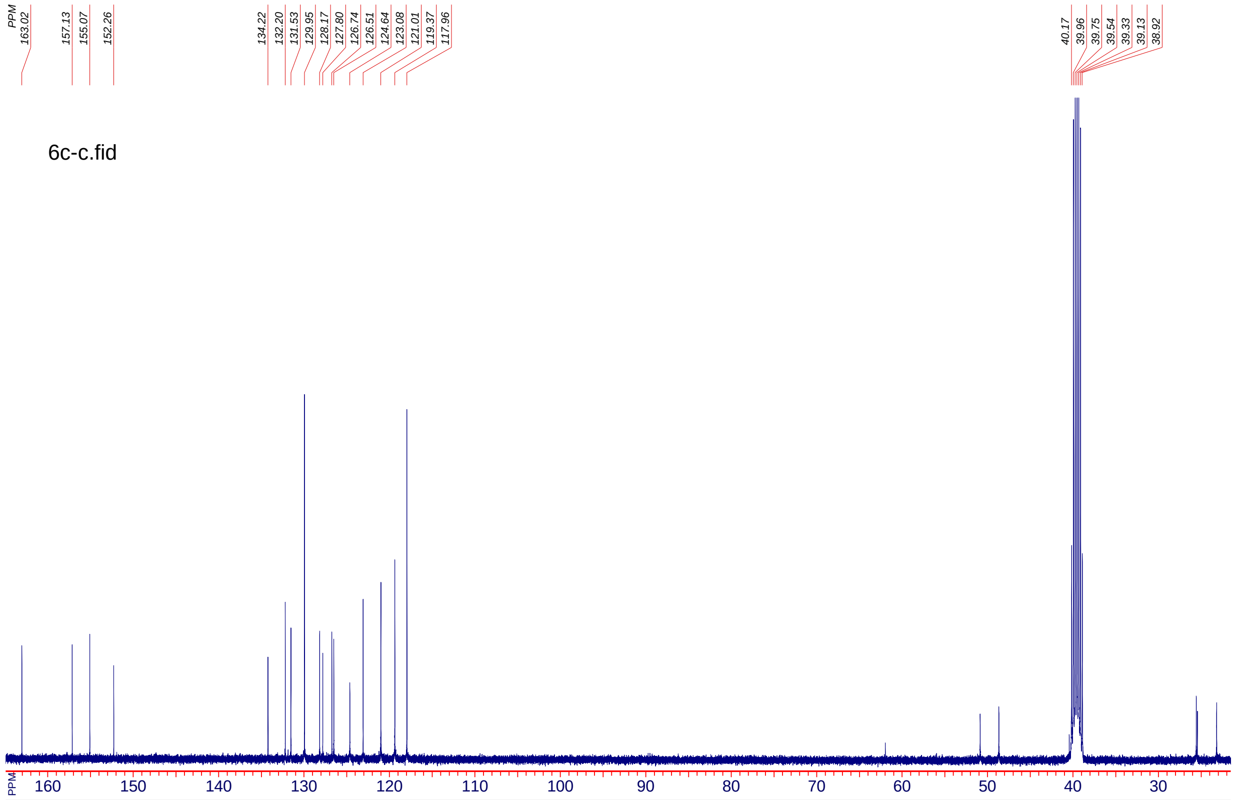

**File name: 6c-c.fid**	Operator:	SF: 100.6117 MHz	NSC: 0	PW: 4.30 usec, RG: 36	SI: 131072
Date: 04-Sep-2020	Solvent: dmso	SW: 28409 Hz	TE: 294 K	AQ: 2.25 sec, RD: 0.00 sec	dmso 0122

*3-(4-Methoxyphenyl)-1-[(4-phenoxyphenylcarbamoyl)-methyl]-6,7-dihydro-5H-pyrrolo[1,2-a]imidazol-1-ium chloride (**6d**)*

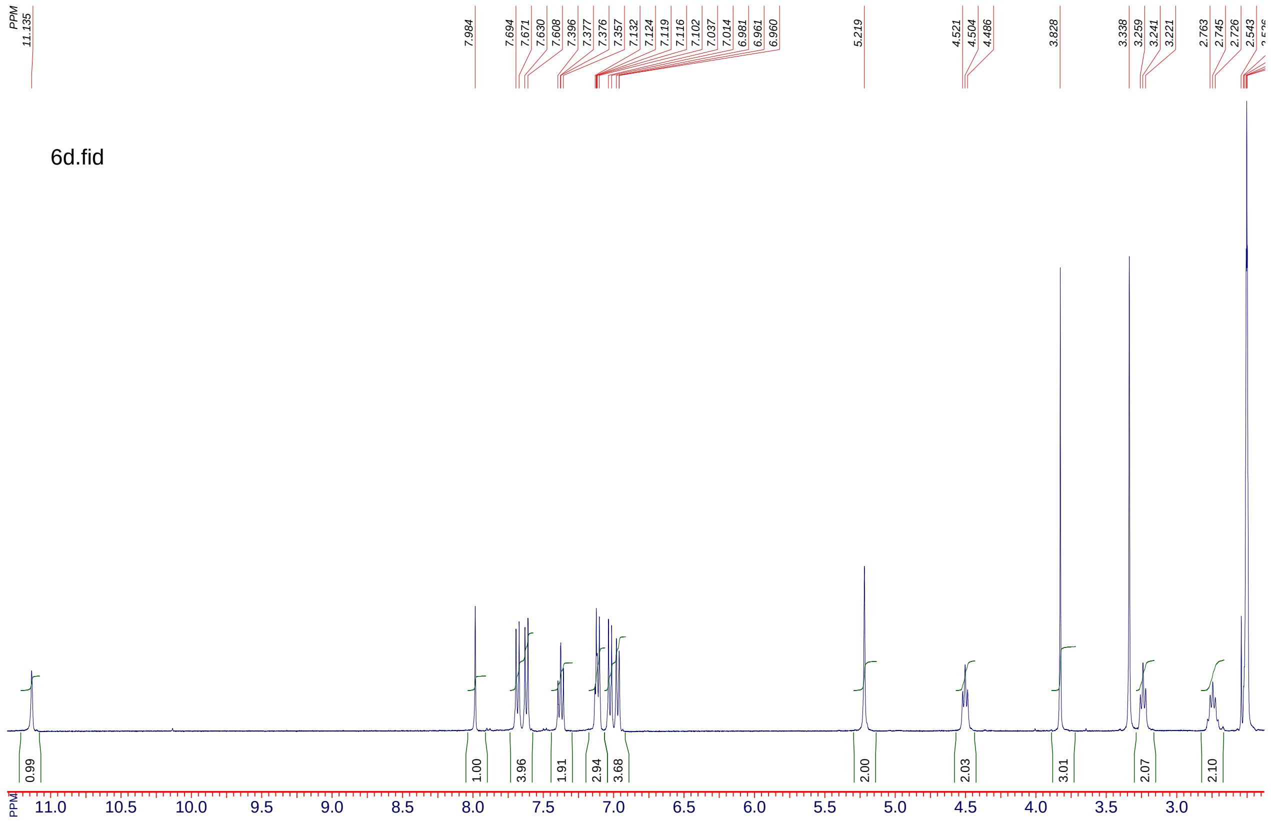

**File name: 6d.fid**	Operator:	SF: 400.0773 MHz	NSC: 0	PW: 5.30 usec, RG: 30	SI: 32768
Date: 13-Jul-2020	Solvent: dmso	SW: 8013 Hz	TE: 293 K	AQ: 2.04 sec, RD: 0.00 sec	dmso new

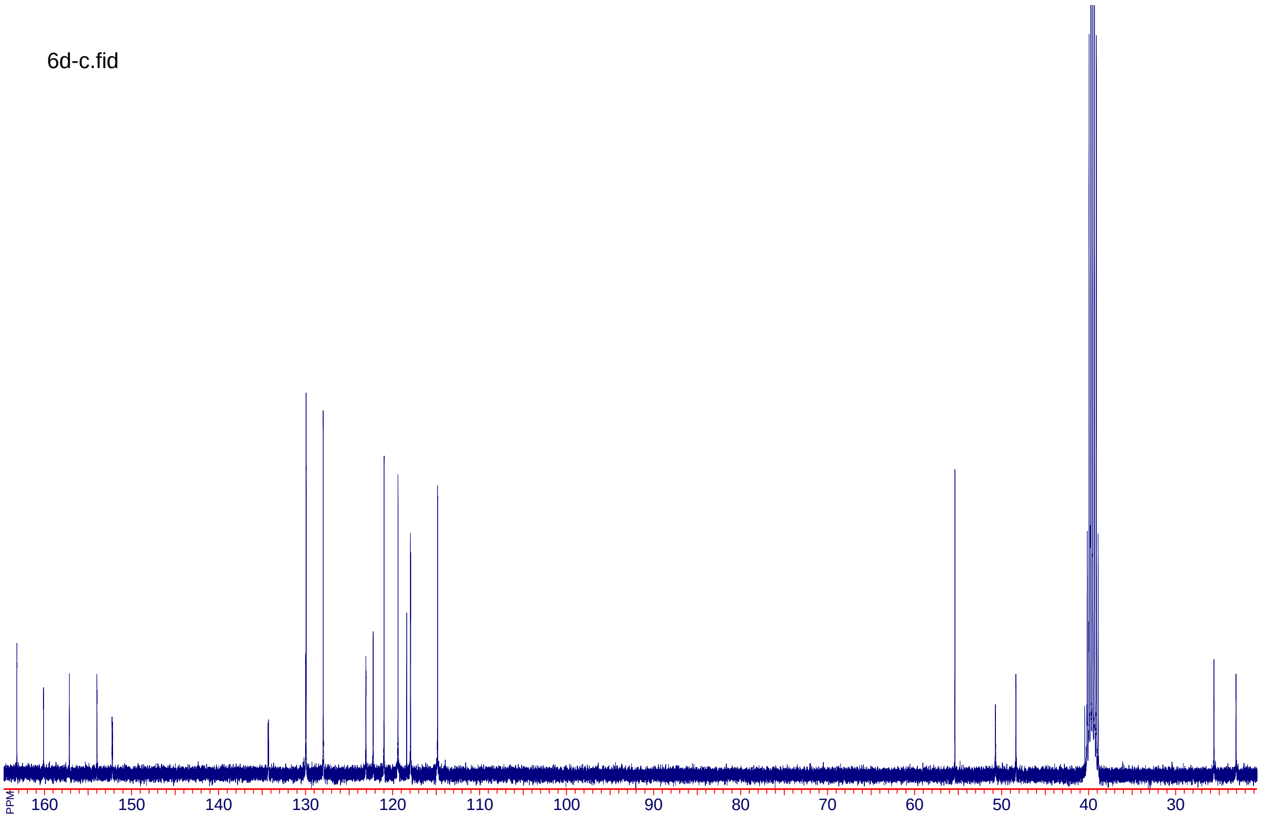

**File name: 6d-c.fid**	Operator:	SF: 100.6117 MHz	NSC: 0	PW: 4.30 usec, RG: 36	SI: 131072
Date: 03-Sep-2020	Solvent: dmso	SW: 28409 Hz	TE: 294 K	AQ: 2.25 sec, RD: 0.00 sec	dmso 0122

*1-[(4-Phenoxyphenylcarbamoyl)-methyl]-3-p-tolyl-6,7-dihydro-5H-pyrrolo[1,2-a]imidazol-1-ium chloride (**6e**)*

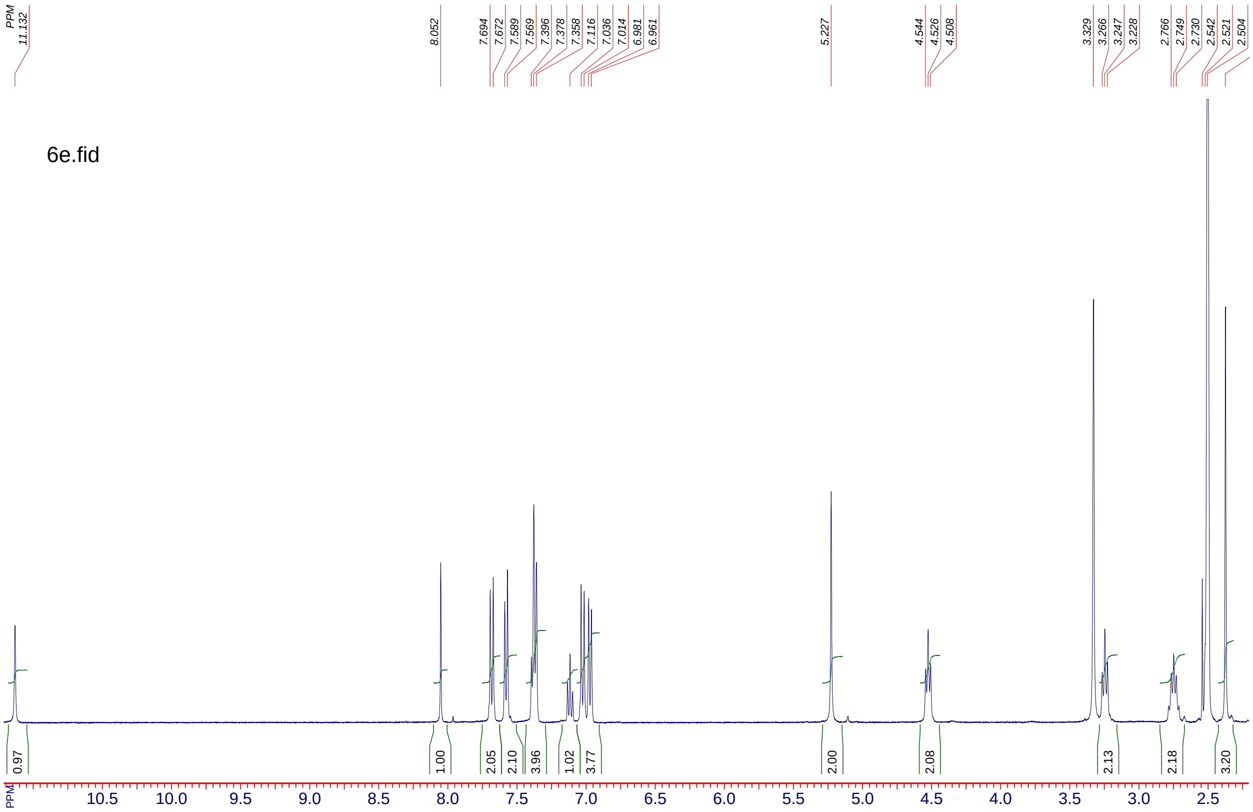

**File name: 6e.fid**	Operator:	SF: 400.0773 MHz	NSC: 0	PW: 5.30 usec, RG: 30	SI: 32768
Date: 13-Jul-2020	Solvent: dmso	SW: 8013 Hz	TE: 293 K	AQ: 2.04 sec, RD: 0.00 sec	dmso new

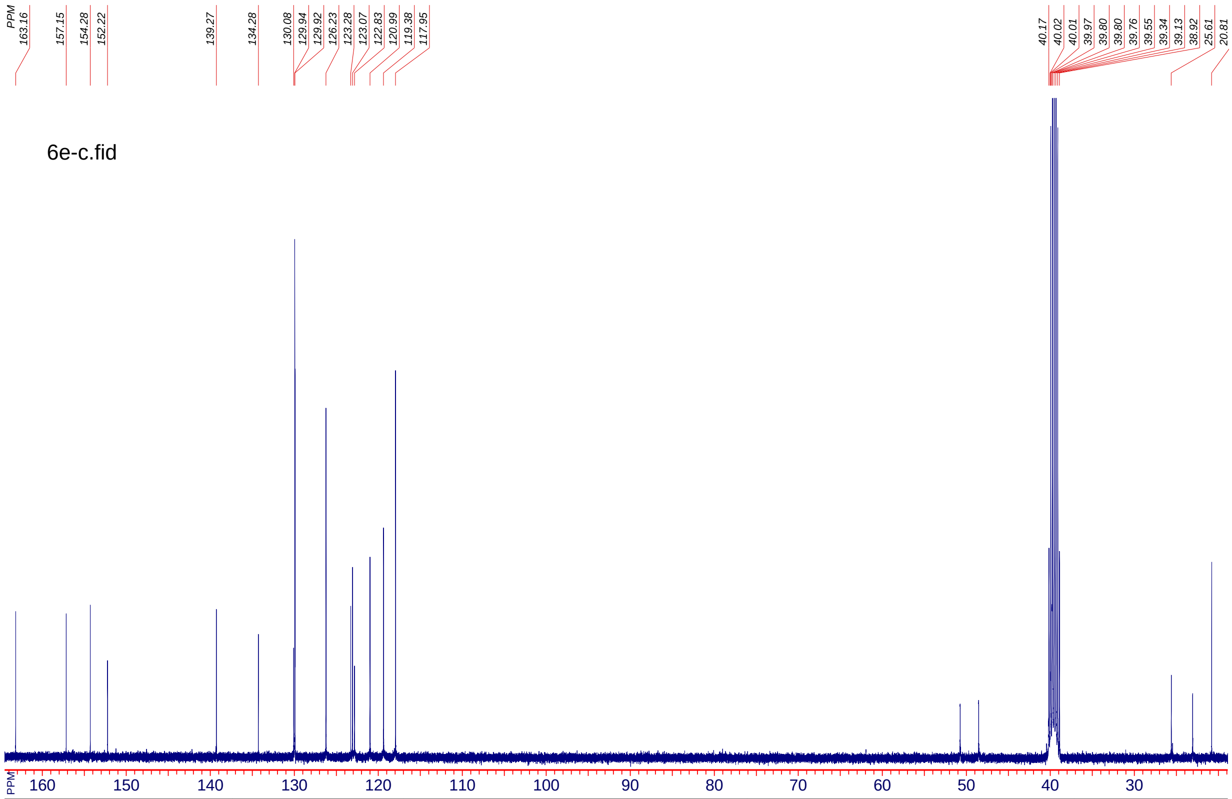

**File name: 6e-c.fid**	Operator:	SF: 100.6117 MHz	NSC: 0	PW: 4.30 usec, RG: 38	SI: 131072
Date: 08-Sep-2020	Solvent: dmso	SW: 28409 Hz	TE: 294 K	AQ: 2.25 sec, RD: 0.00 sec	dmso 0122

1-(3,4-Dichlorobenzyl)-3-phenyl-6,7,8,9-tetrahydro-5H-imidazo[1,2-a]azepin-1-ium bromide (**9**)

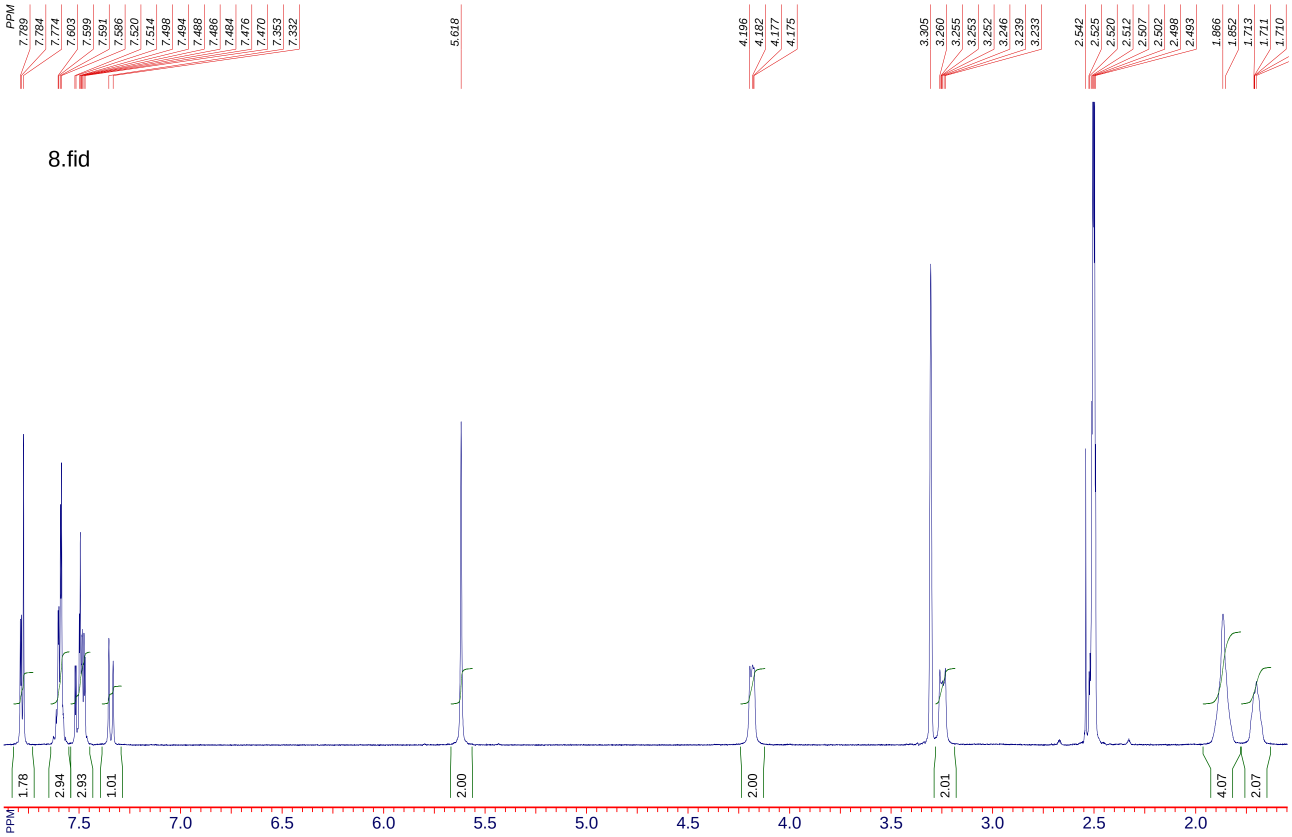

File name: 8.fid	Operator:	SF: 400.0773 MHz	NSC: 0	PW: 5.30 usec, RG: 30	SI: 32768
Date: 13-Jul-2020	Solvent: dmso	SW: 8013 Hz	TE: 293 K	AQ: 2.04 sec, RD: 0.00 sec	dmso new

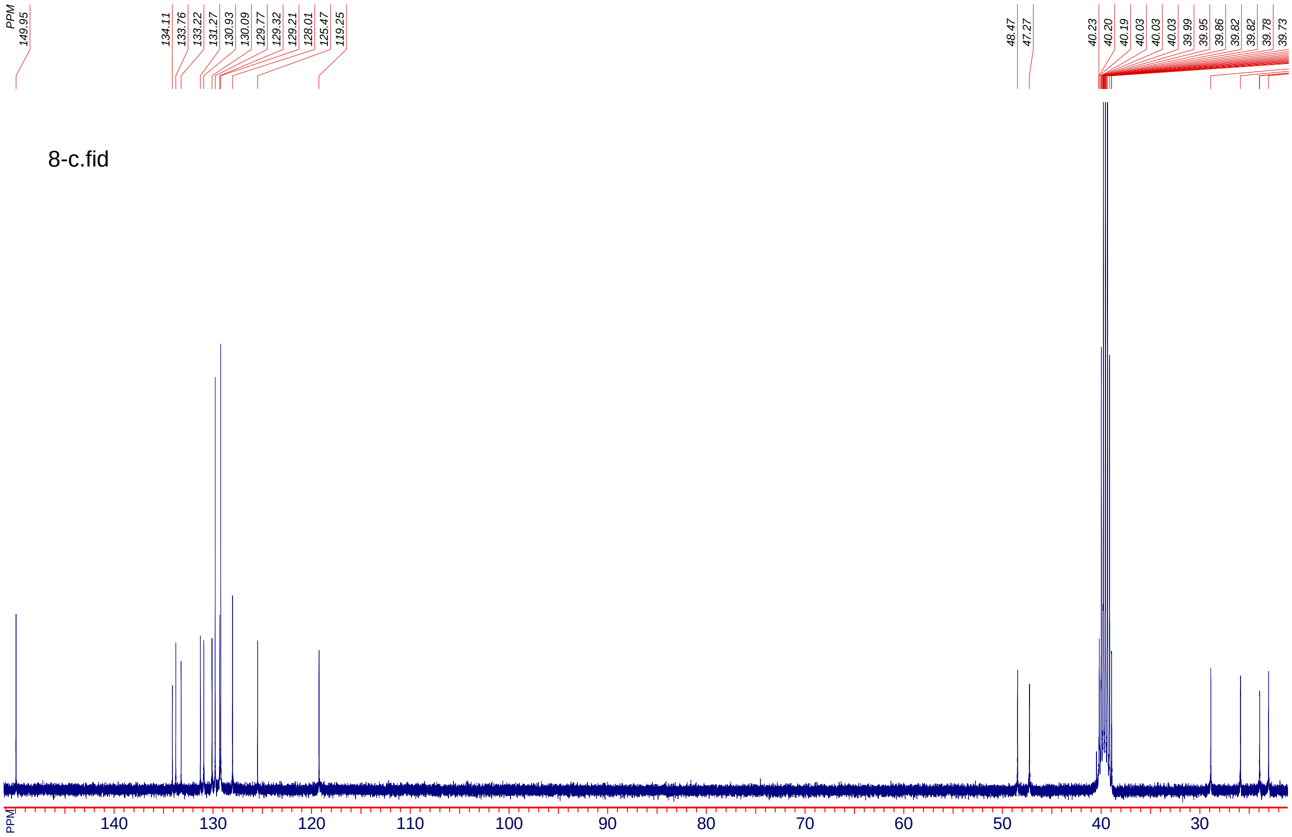

File name: 8-c.fid	Operator:	SF: 100.6117 MHz	NSC: 0	PW: 4.30 usec, RG: 38	SI: 131072
Date: 14-Sep-2020	Solvent: dmso	SW: 28409 Hz	TE: 294 K	AQ: 2.25 sec, RD: 0.00 sec	dmso 0122

*1-[2-(2-Difluoromethoxyphenyl)-2-oxoethyl]-3-phenyl-6,7,8,9-tetrahydro-5H-imidazo[1,2-a]azepin-1-ium bromide (**10a**)*

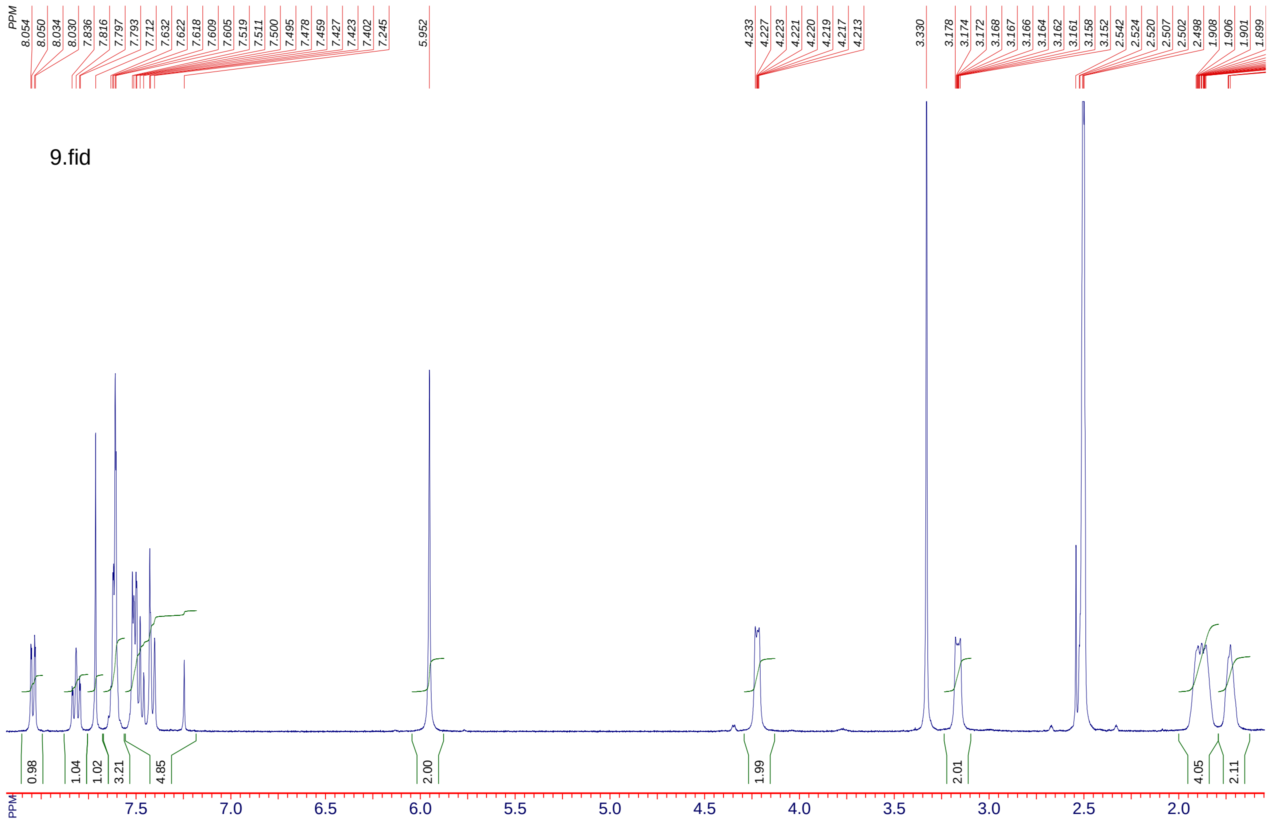

**File name: 9.fid**	Operator:	SF: 400.0773 MHz	NSC: 0	PW: 5.30 usec, RG: 30	SI: 32768
Date: 13-Jul-2020	Solvent: dmso	SW: 8013 Hz	TE: 293 K	AQ: 2.04 sec, RD: 0.00 sec	dmso new

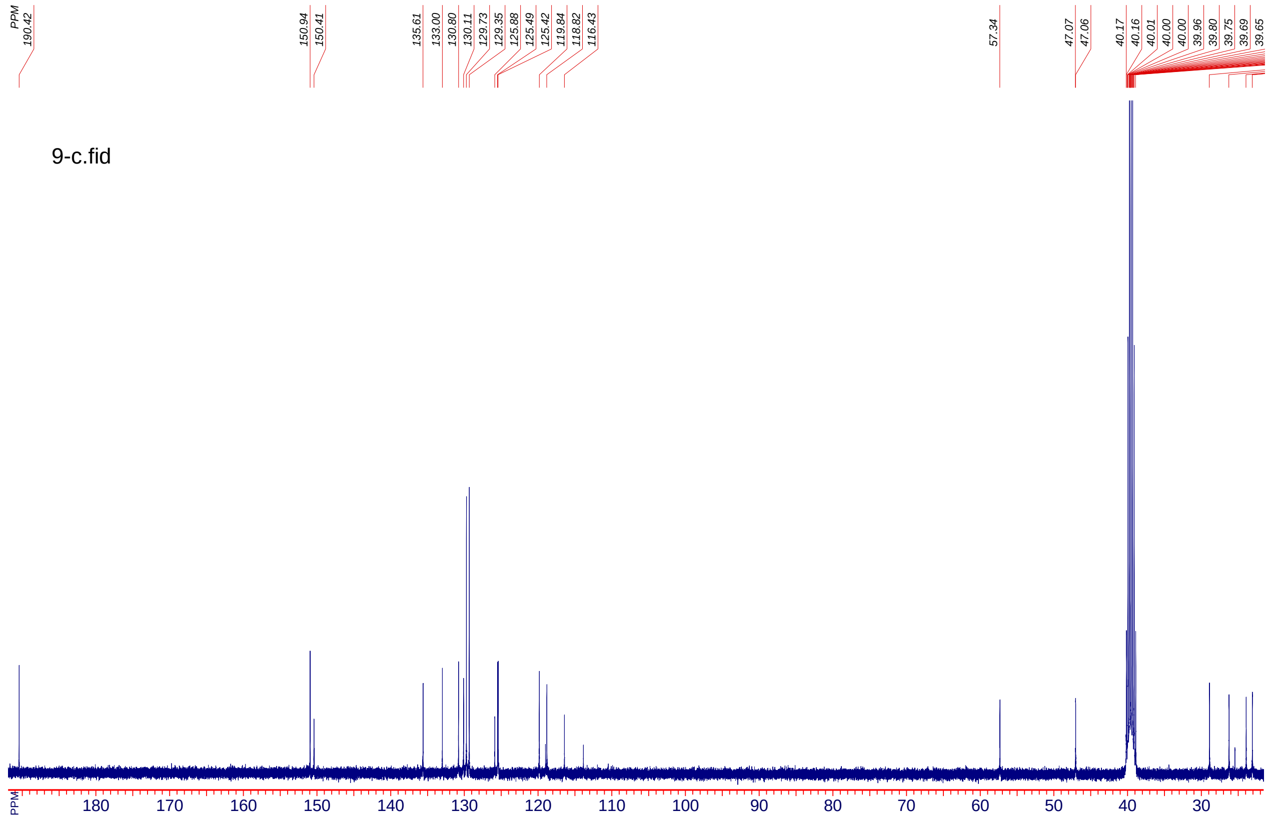

**File name: 9-c.fid**	Operator:	SF: 100.6117 MHz	NSC: 0	PW: 4.30 usec, RG: 36	SI: 131072
Date: 01-Sep-2020	Solvent: dmso	SW: 28409 Hz	TE: 294 K	AQ: 2.25 sec, RD: 0.00 sec	dmso 0122

*1-[2-(4-Isopropylphenyl)-2-oxoethyl]-3-phenyl-6,7,8,9-tetrahydro-5H-imidazo[1,2-a]azepin-1-ium bromide (**10b**)*

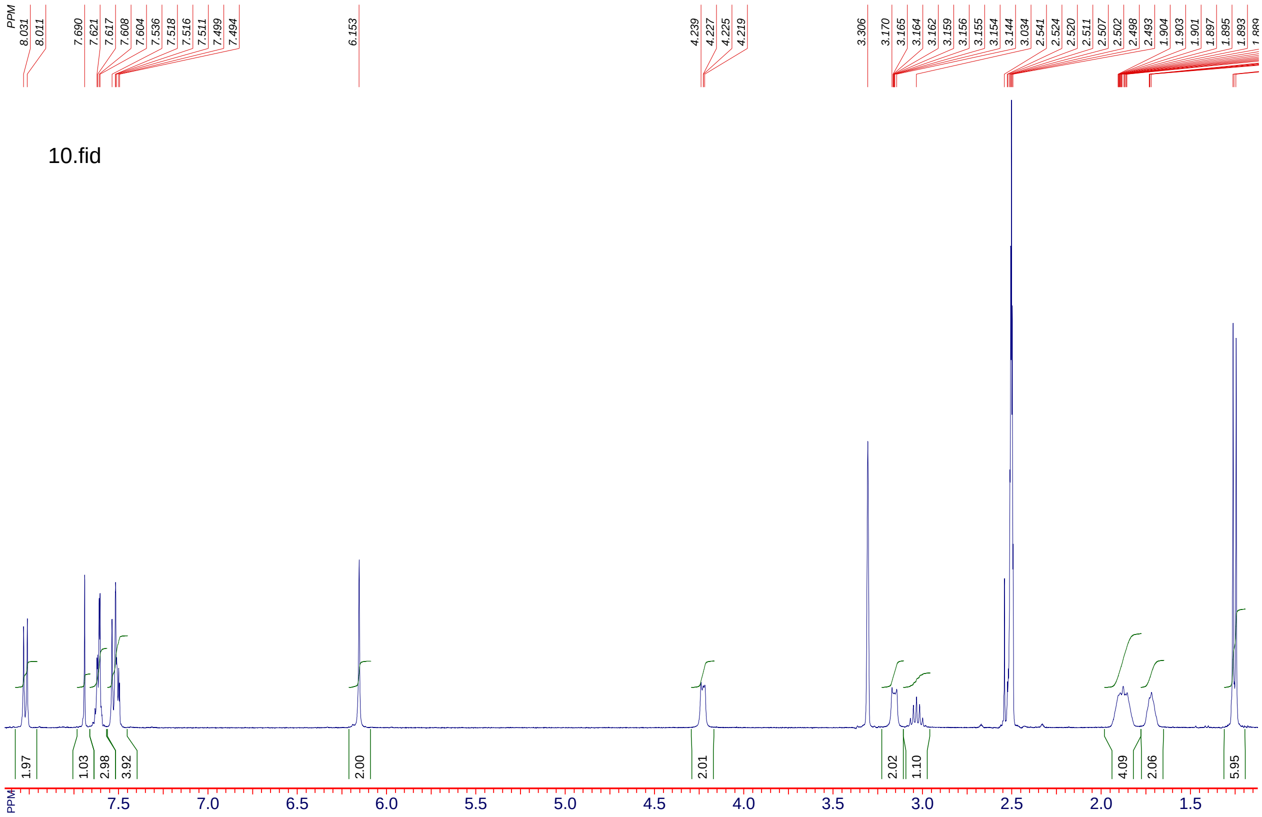

**File name: 10.fid**	Operator:	SF: 400.0773 MHz	NSC: 0	PW: 5.30 usec, RG: 30	SI: 32768
Date: 13-Jul-2020	Solvent: dmso	SW: 8013 Hz	TE: 293 K	AQ: 2.04 sec, RD: 0.00 sec	dmso new

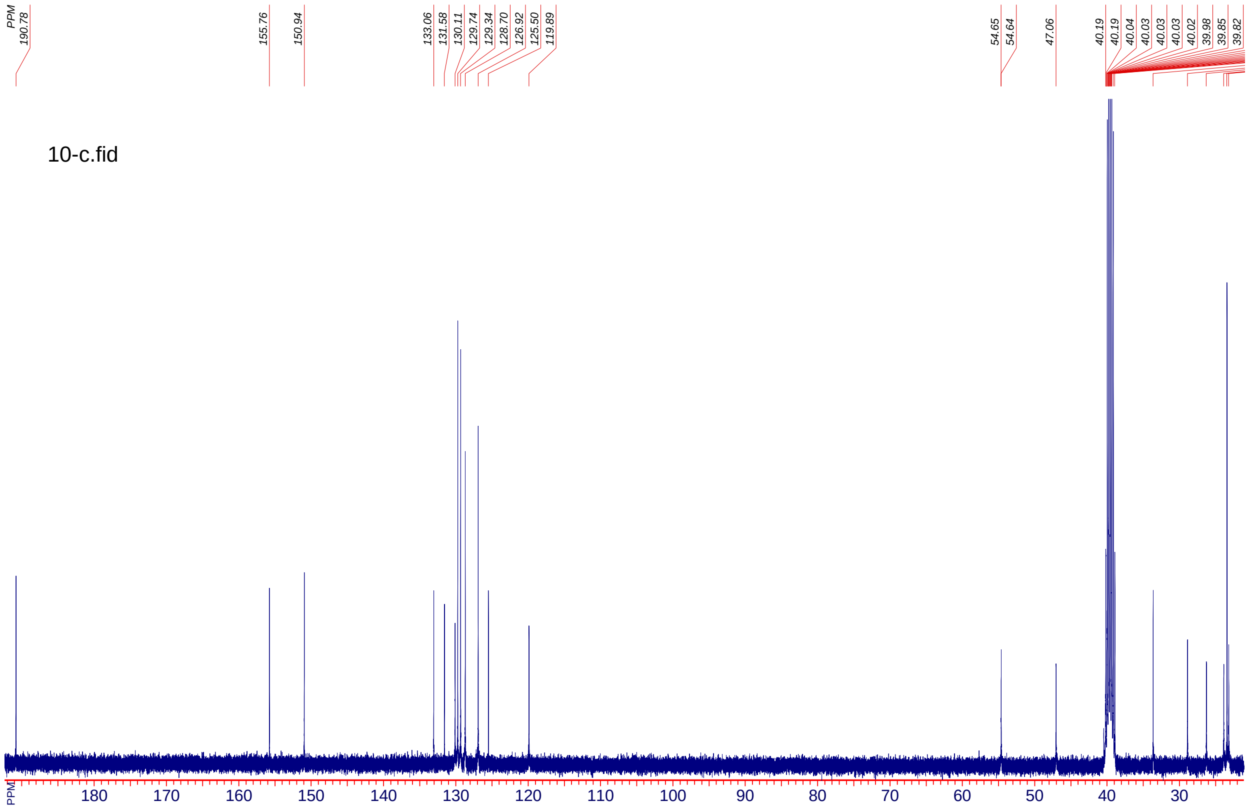

**File name: 10-c.fid**	Operator:	SF: 100.6117 MHz	NSC: 0	PW: 4.30 usec, RG: 38	SI: 131072
Date: 18-Sep-2020	Solvent: dmso	SW: 28409 Hz	TE: 294 K	AQ: 2.25 sec, RD: 0.00 sec	dmso 0122

*1-[2-Oxo-2-(4-phenoxyphenyl)-ethyl]-3-phenyl-6,7,8,9-tetrahydro-5H-imidazo[1,2-a]azepin-1-ium bromide (**10c**)*

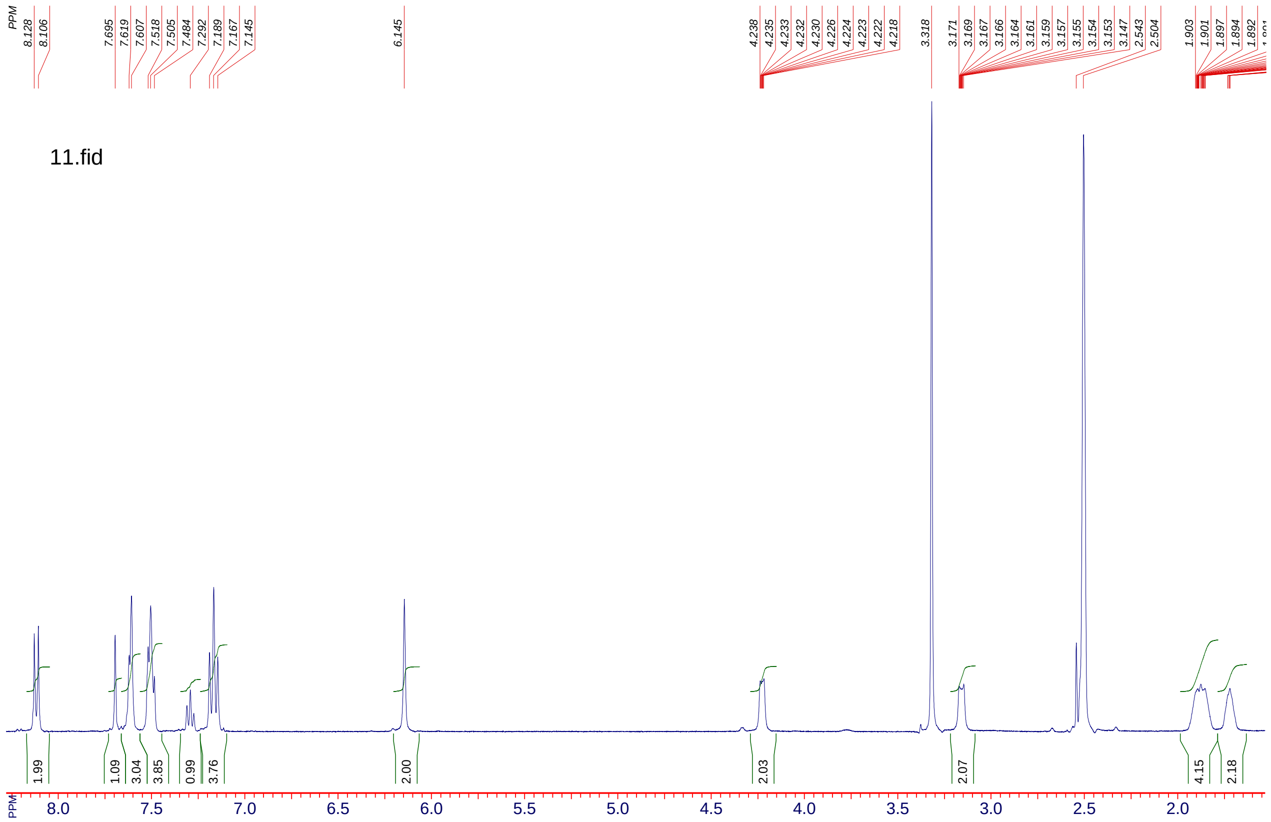

File name: 11.fid	Operator:	SF: 400.0773 MHz	NSC: 0	PW: 5.30 usec, RG: 30	SI: 32768
Date: 13-Jul-2020	Solvent: dmso	SW: 8013 Hz	TE: 293 K	AQ: 2.04 sec, RD: 0.00 sec	dmso new

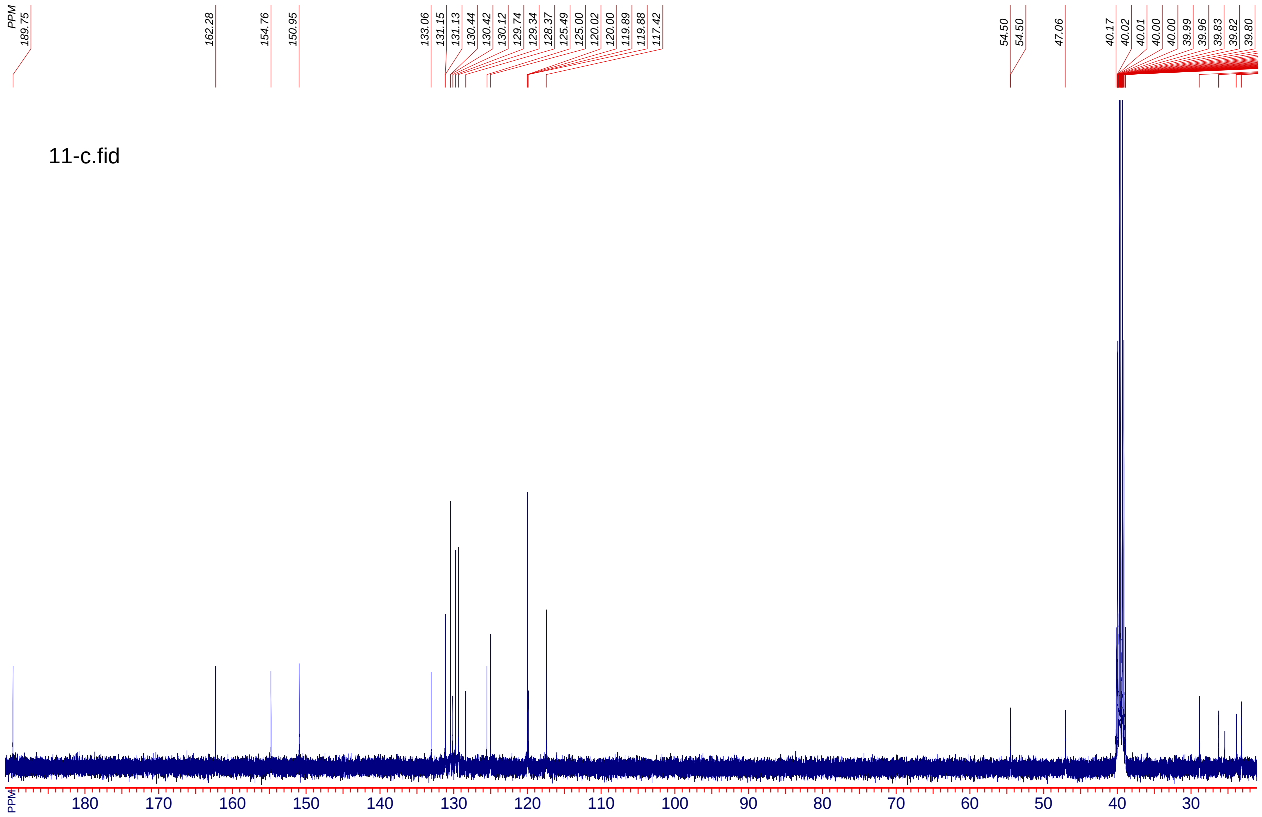

File name: 11-c.fid	Operator:	SF: 100.6117 MHz	NSC: 0	PW: 4.30 usec, RG: 36	SI: 131072
Date: 12-Sep-2020	Solvent: dmso	SW: 28409 Hz	TE: 294 K	AQ: 2.25 sec, RD: 0.00 sec	dmso 0122

*1-[2-(4-Cyclohexylphenyl)-2-oxoethyl]-3-(4-fluorophenyl)-6,7,8,9-tetrahydro-5H-imidazo[1,2-a]azepin-1-ium bromide (**10d**)*

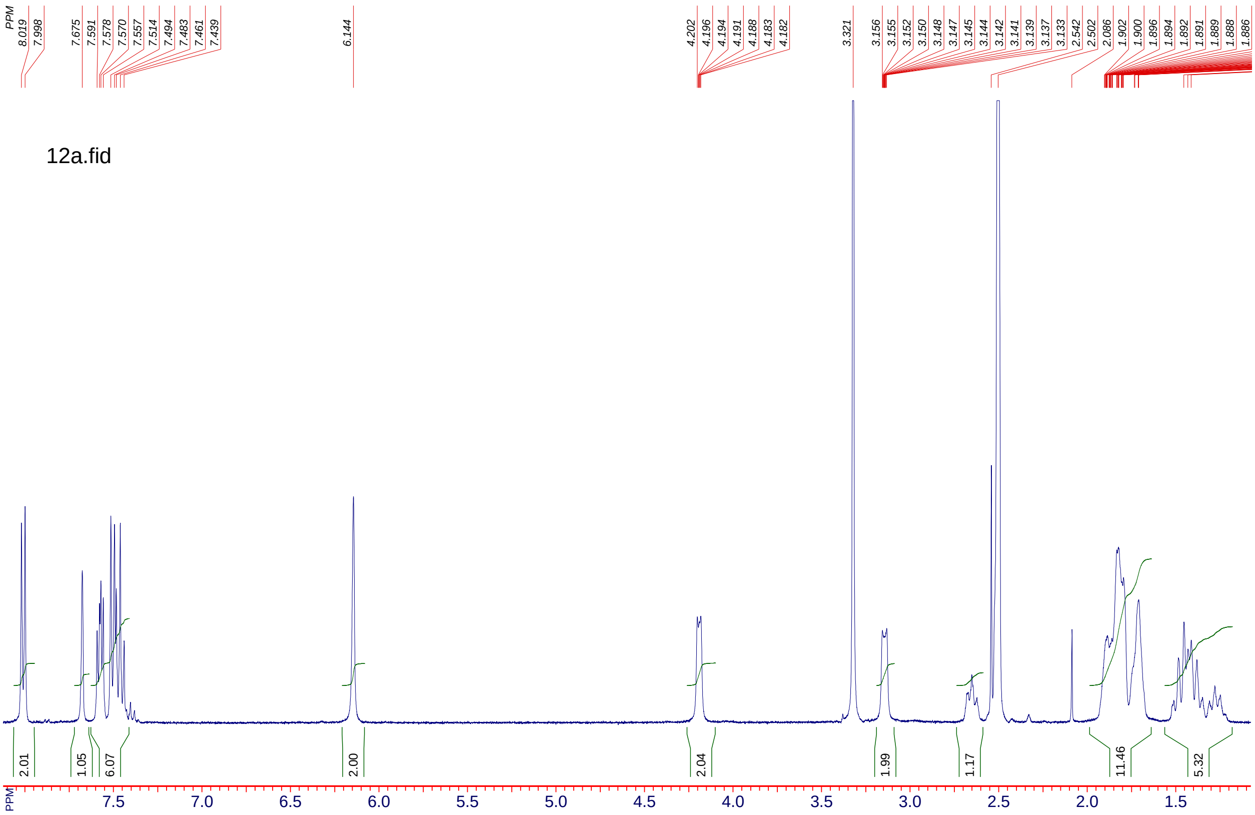

**File name: 12a.fid**	Operator:	SF: 400.0773 MHz	NSC: 0	PW: 5.30 usec, RG: 30	SI: 32768
Date: 13-Jul-2020	Solvent: dmso	SW: 8013 Hz	TE: 293 K	AQ: 2.04 sec, RD: 0.00 sec	dmso new

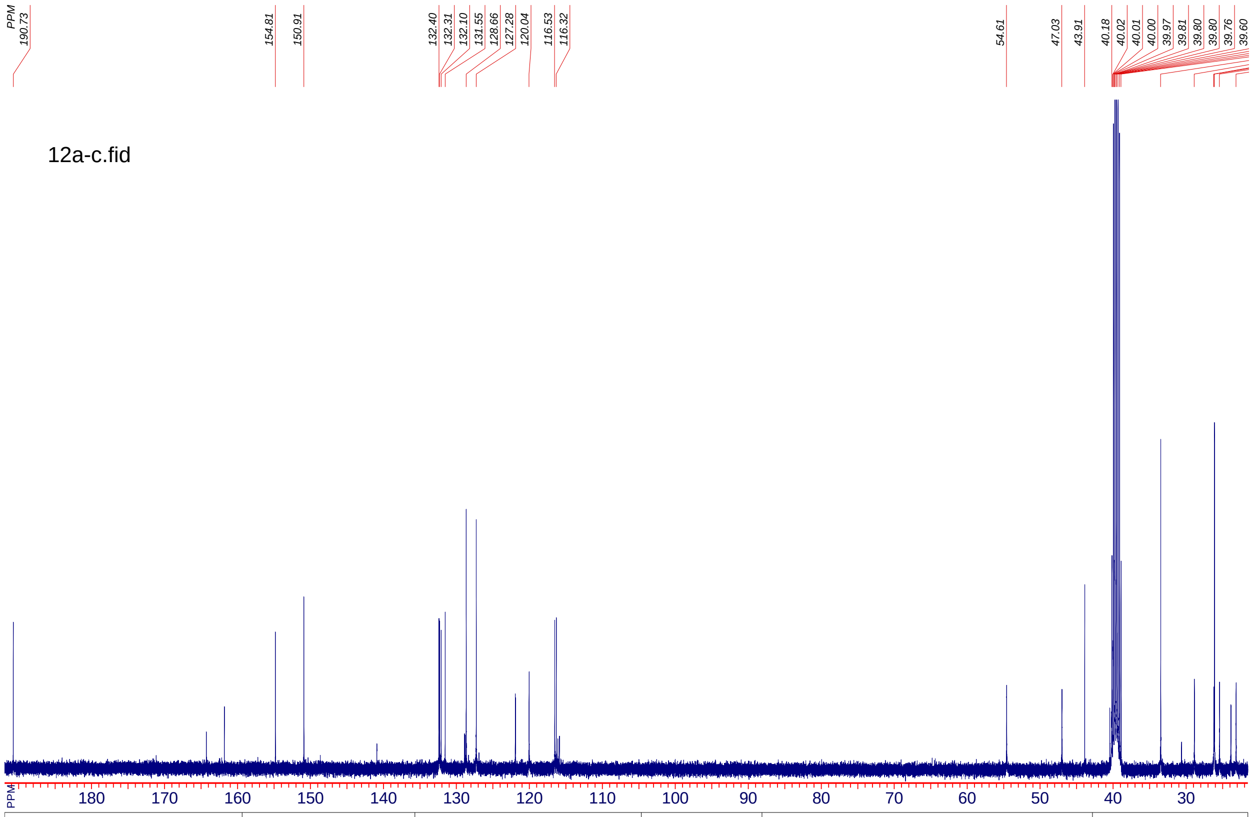

**File name: 12a-c.fid**	Operator:	SF: 100.6117 MHz	NSC: 0	PW: 4.30 usec, RG: 36	SI: 131072
Date: 02-Sep-2020	Solvent: dmso	SW: 28409 Hz	TE: 294 K	AQ: 2.25 sec, RD: 0.00 sec	dmso 0122

3-(4-Chlorophenyl)-1-[2-(4-cyclohexylphenyl)-2-oxoethyl]-6,7,8,9-tetrahydro-5H-imidazo[1,2-a]azepin-1-ium bromide (**10e**)

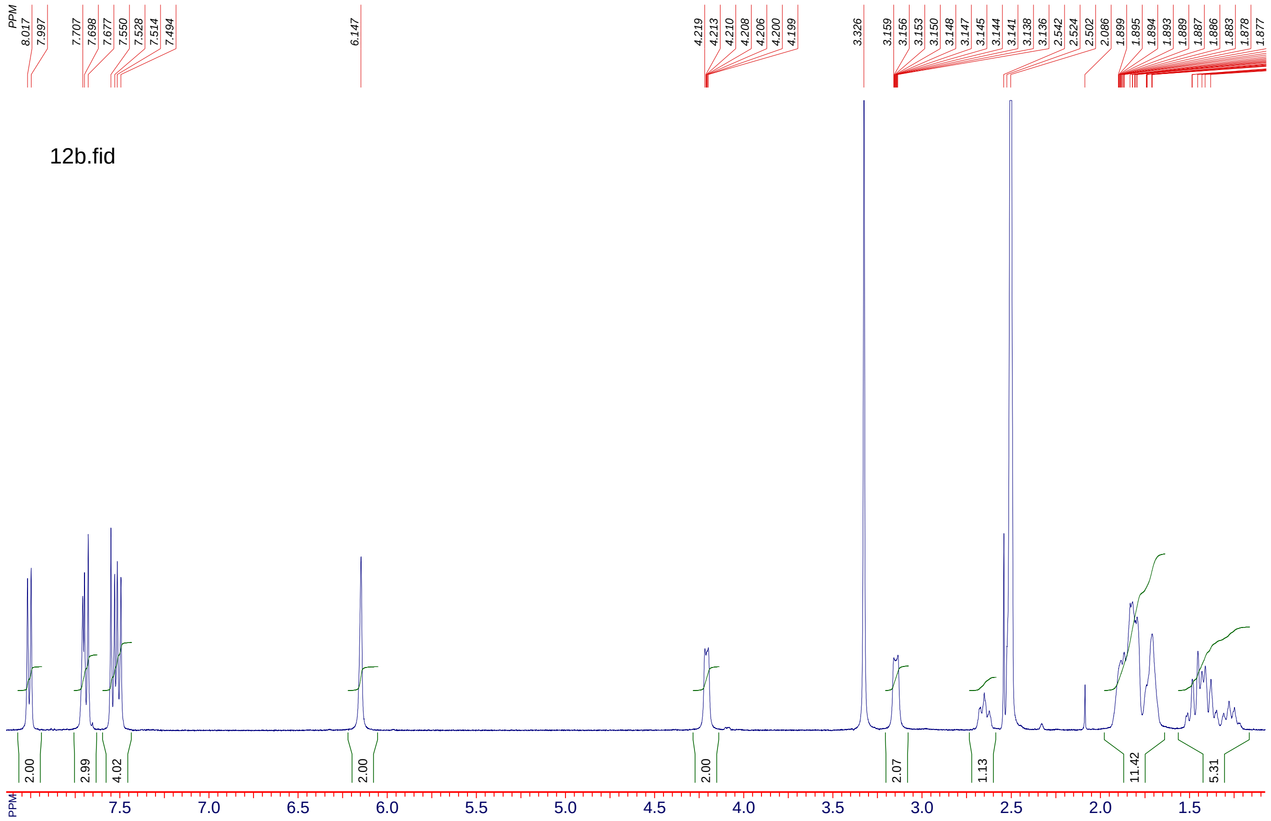

File name: 12b.fid	Operator:	SF: 400.0773 MHz	NSC: 0	PW: 5.30 usec, RG: 30	SI: 32768
Date: 13-Jul-2020	Solvent: dmso	SW: 8013 Hz	TE: 293 K	AQ: 2.04 sec, RD: 0.00 sec	dmso new

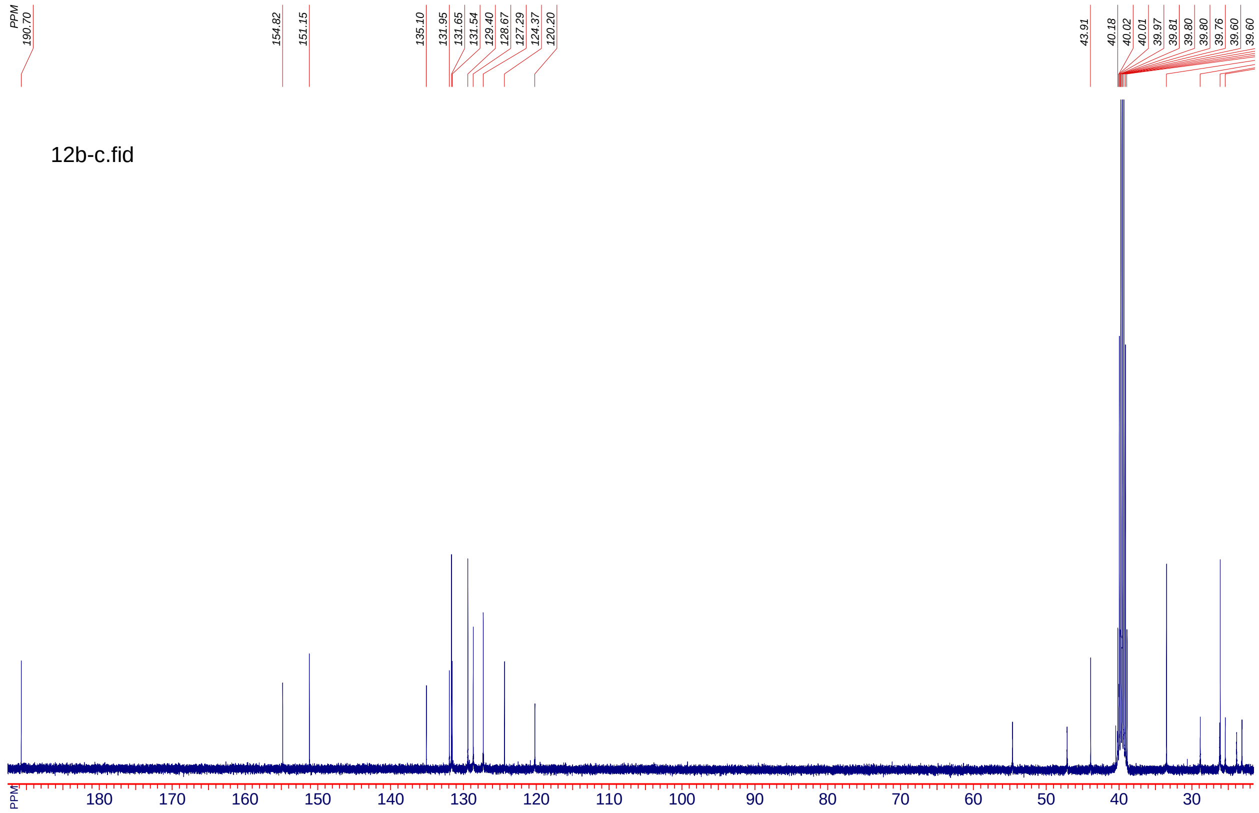

File name: 12b-c.fid	Operator:	SF: 100.6117 MHz	NSC: 0	PW: 4.30 usec, RG: 38	SI: 131072
Date: 17-Sep-2020	Solvent: dmso	SW: 28409 Hz	TE: 294 K	AQ: 2.25 sec, RD: 0.00 sec	dmso 0122

*1-[2-(4-Bromophenyl)-2-oxoethyl]-3-(3,4-dichlorophenyl)-6,7,8,9-tetrahydro-5H-imidazo[1,2-a]azepin-1-ium bromide (**10f**)*

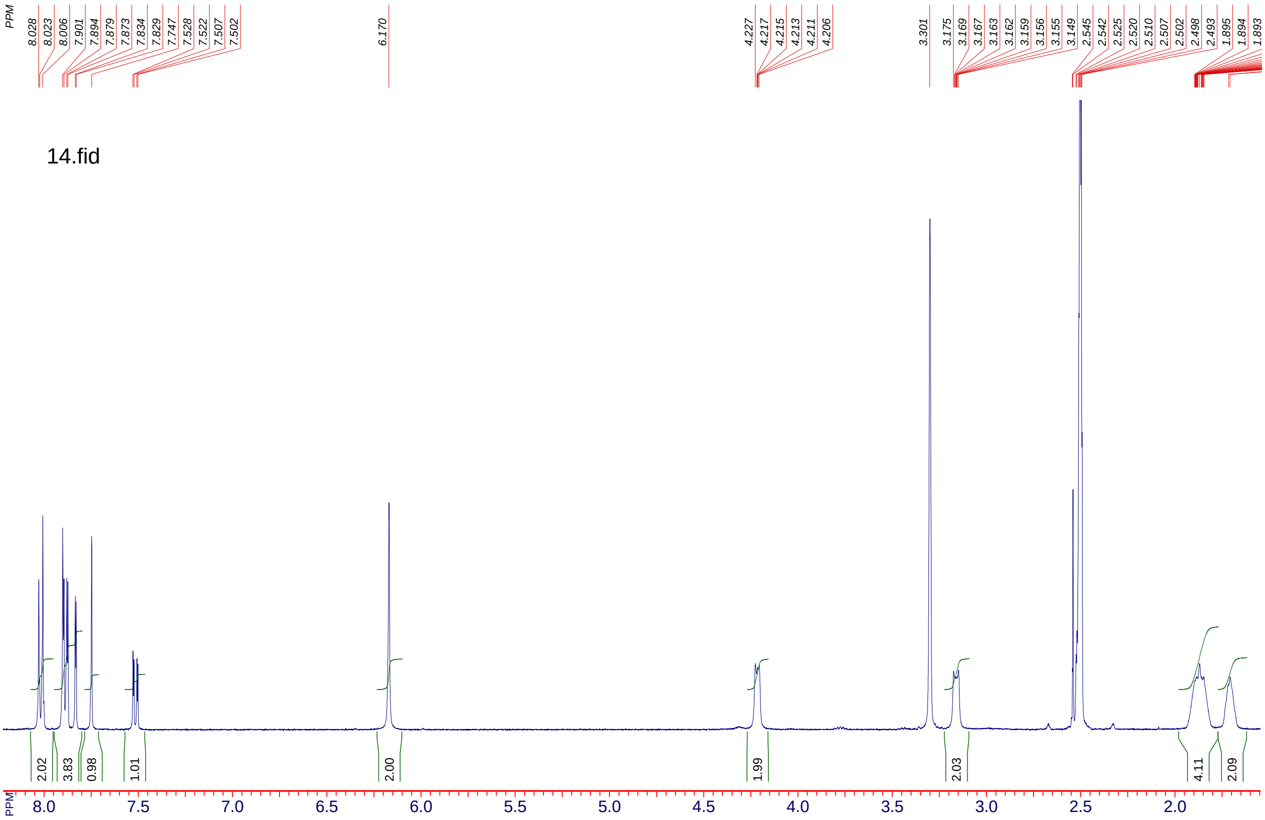

File name: 14.fid	Operator:	SF: 400.0773 MHz	NSC: 0	PW: 5.30 usec, RG: 30	SI: 32768
Date: 13-Jul-2020	Solvent: dmso	SW: 8013 Hz	TE: 293 K	AQ: 2.04 sec, RD: 0.00 sec	dmso new

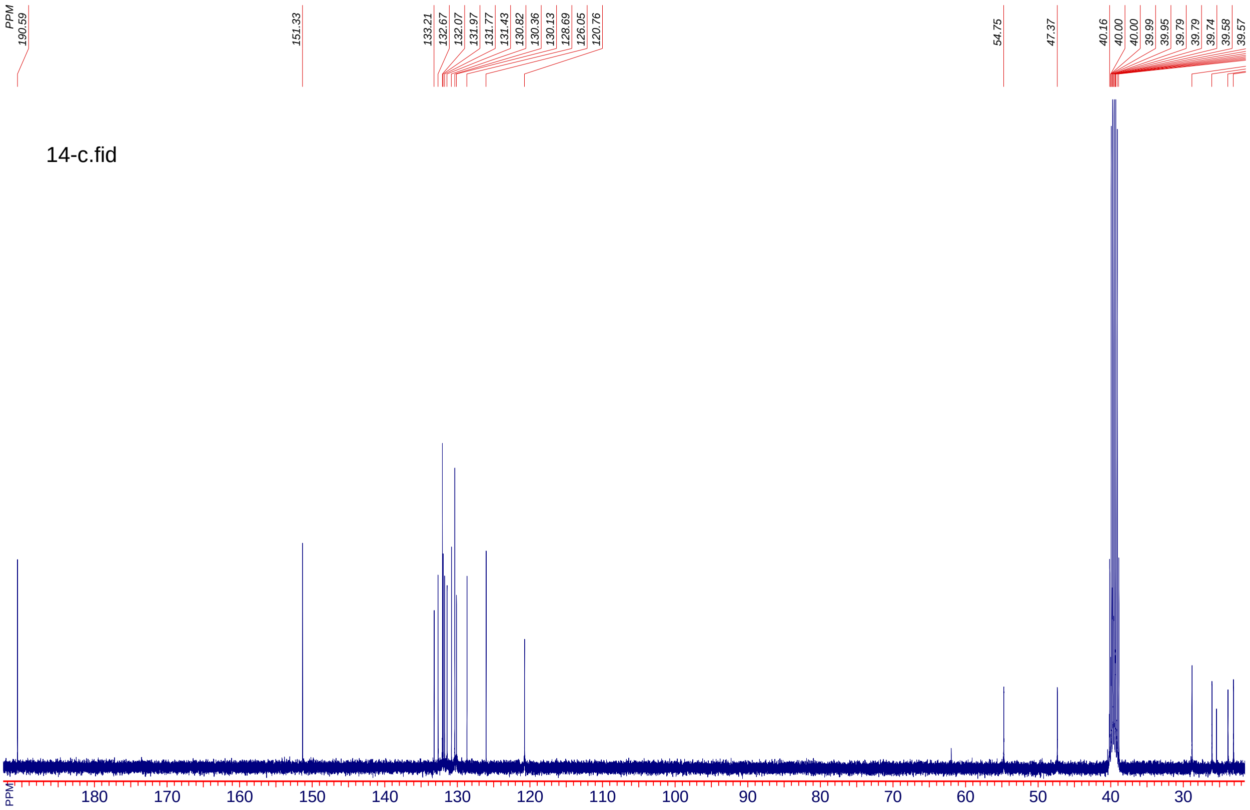

File name: 14-c.fid	Operator:	SF: 100.6117 MHz	NSC: 0	PW: 4.30 usec, RG: 36	SI: 131072
Date: 31-Aug-2020	Solvent: dmso	SW: 28409 Hz	TE: 294 K	AQ: 2.25 sec, RD: 0.00 sec	dmso 0122

*3-(3,4-Dichlorophenyl)-1-[2-(4-methoxyphenyl)-2-oxoethyl]-6,7,8,9-tetrahydro-5H-imidazo[1,2-a]azepin-1-ium bromide (**10h**)*

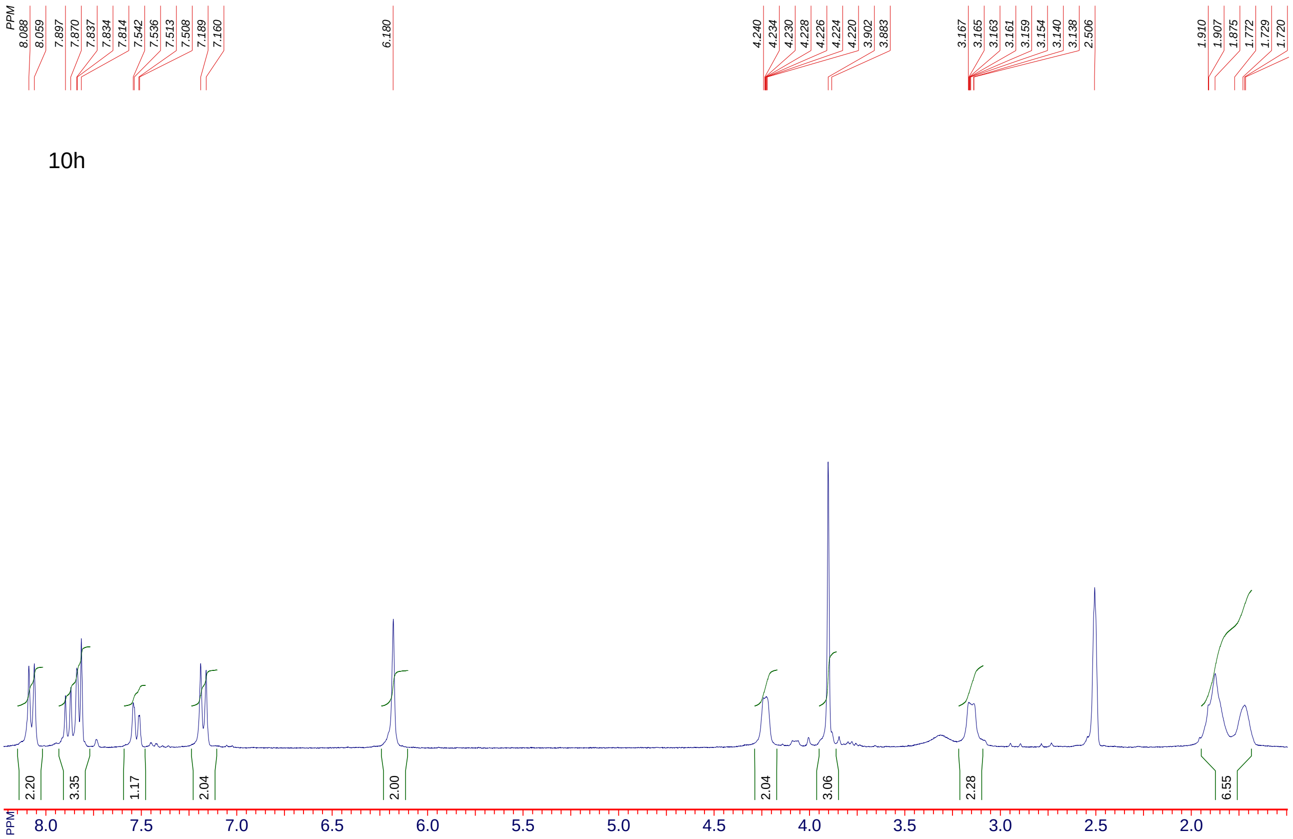

**File name: 10h**	Operator:	SF: 299.9450 MHz	NSC: -1	PW: 7.00 usec, RG: 0	SI: 16384
Date:	Solvent:	SW: 5099 Hz	TE: 0 K	AQ: 3.21 sec, RD: 1.50 sec	

*3-(3,4-Dichlorophenyl)-1-[(isopropylphenylcarbamoyl)-methyl]-6,7,8,9-tetrahydro-5H-imidazo[1,2-a]azepin-1-ium chloride (**11**)*

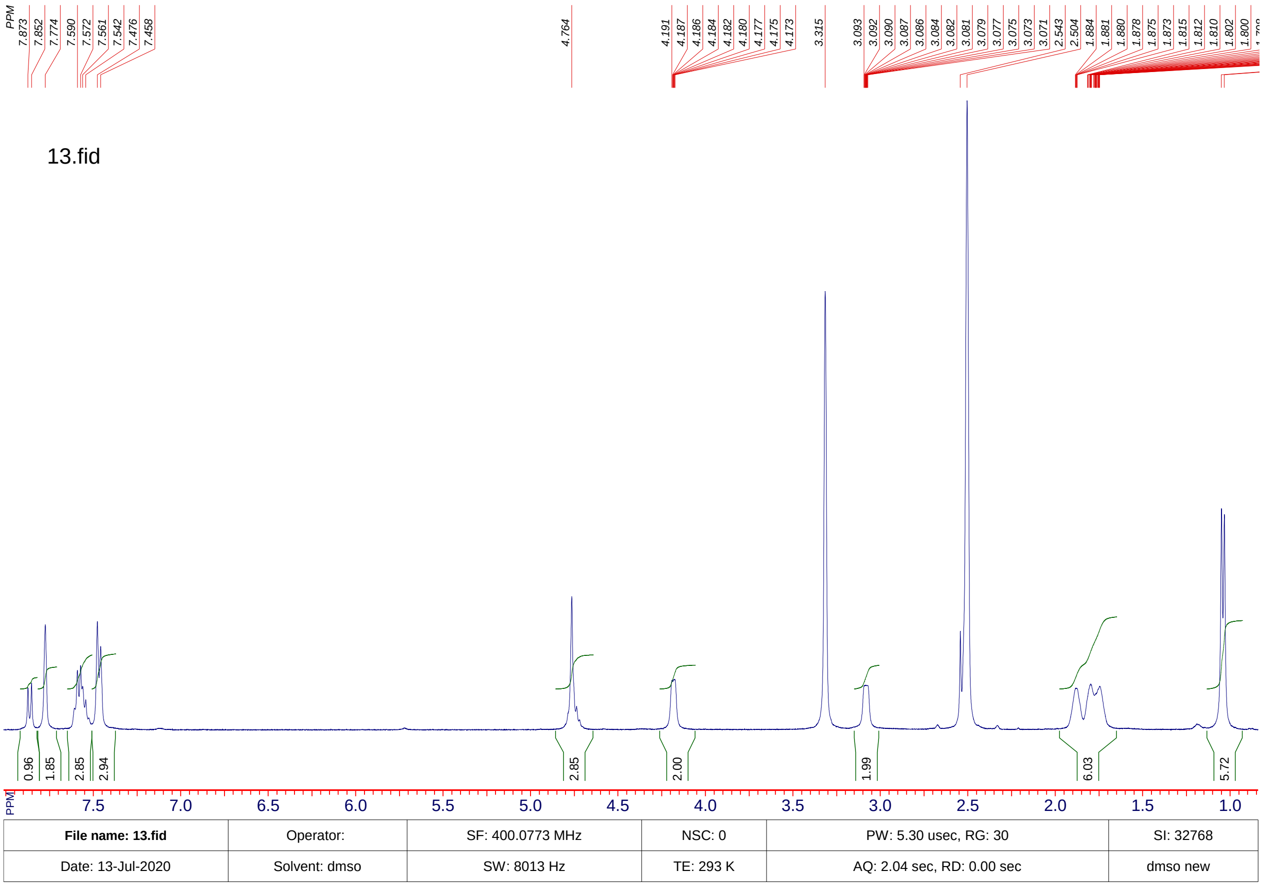

File name: 13.fid	Operator:	SF: 400.0773 MHz	NSC: 0	PW: 5.30 usec, RG: 30	SI: 32768
Date: 13-Jul-2020	Solvent: dmso	SW: 8013 Hz	TE: 293 K	AQ: 2.04 sec, RD: 0.00 sec	dmso new

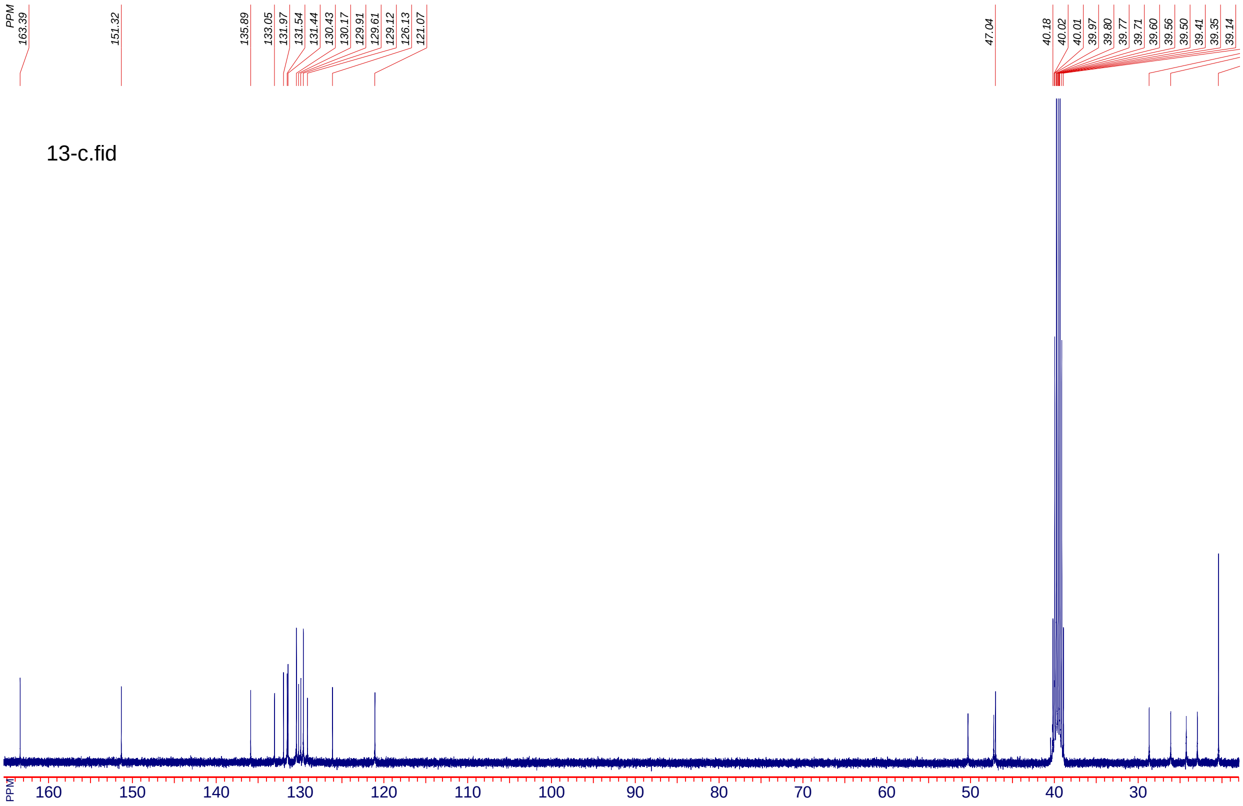

File name: 13-c.fid	Operator:	SF: 100.6117 MHz	NSC: 0	PW: 4.30 usec, RG: 38	SI: 131072
Date: 16-Sep-2020	Solvent: dmso	SW: 28409 Hz	TE: 294 K	AQ: 2.25 sec, RD: 0.00 sec	dmso 0122

*1-(2-Isopropyl-5-methyl-cyclohexyloxycarbonylmethyl)-3-(4-methoxyphenyl)-6,7,8,9-tetrahydro-5H-imidazo[1,2-a]azepin-1-ium chloride (**12**)*

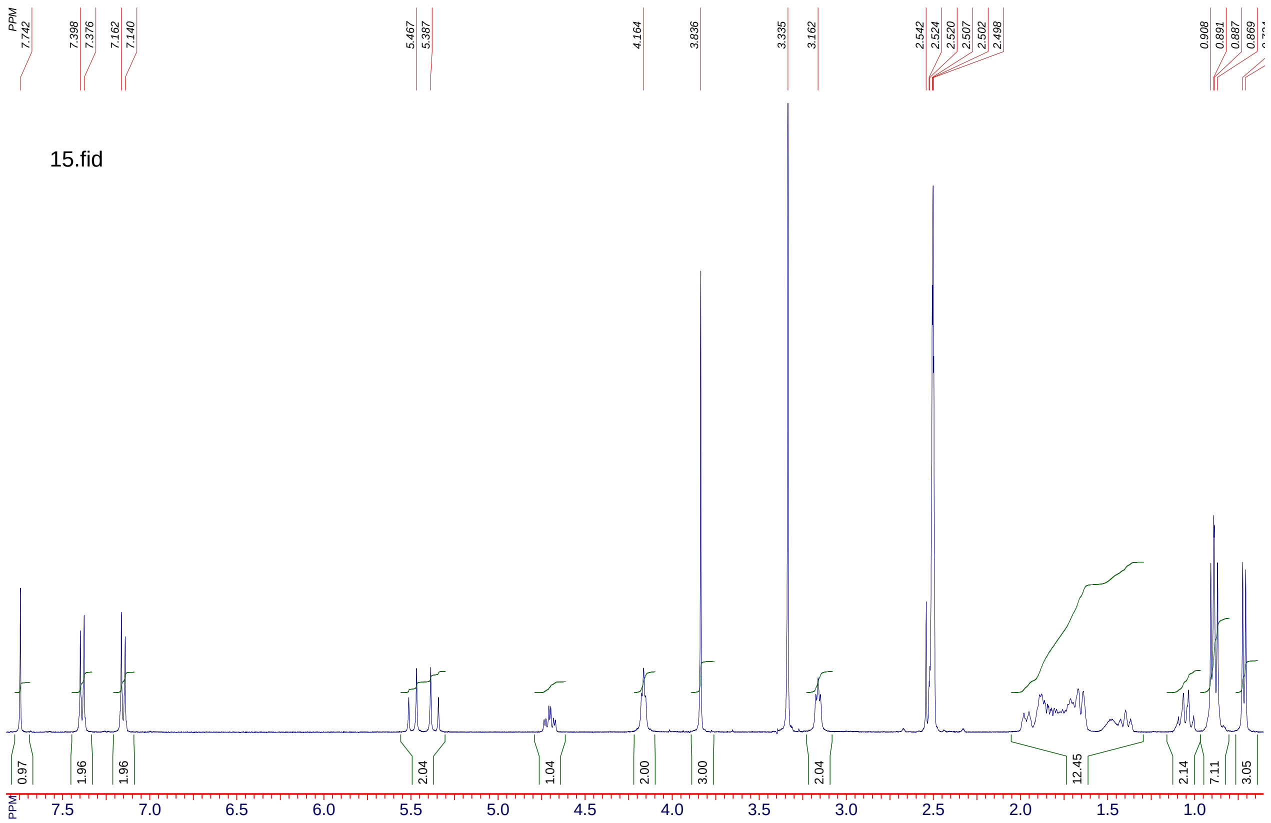

File name: 15.fid	Operator:	SF: 400.0773 MHz	NSC: 0	PW: 5.30 usec, RG: 30	SI: 32768
Date: 13-Jul-2020	Solvent: dmso	SW: 8013 Hz	TE: 293 K	AQ: 2.04 sec, RD: 0.00 sec	dmso new

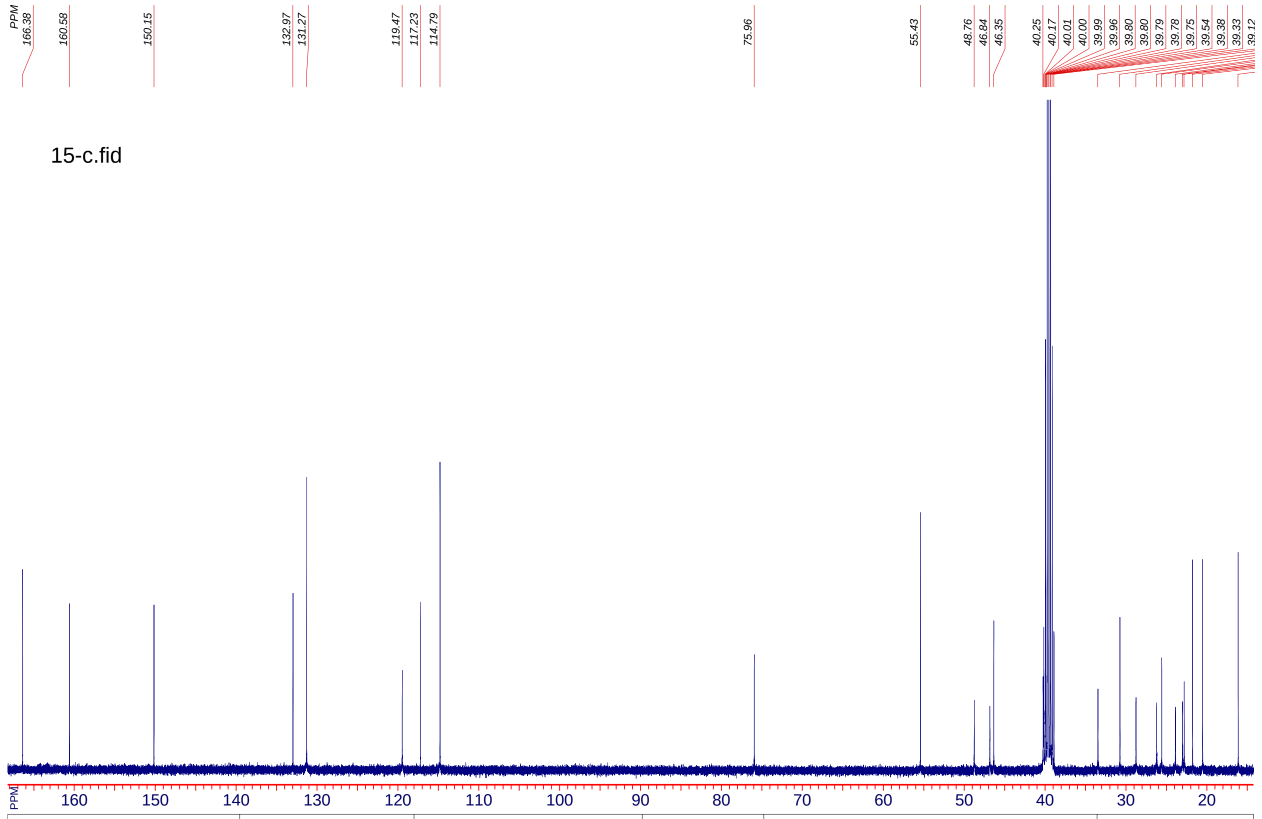

File name: 15-c.fid	Operator:	SF: 100.6117 MHz	NSC: 0	PW: 4.30 usec, RG: 38	SI: 131072
Date: 12-Sep-2020	Solvent: dmso	SW: 28409 Hz	TE: 294 K	AQ: 2.25 sec, RD: 0.00 sec	dmso 0122

4-(3,4-Dichlorophenyl)-1-(4-methoxyphenyl)-5,6,7,8-tetrahydro-2a,4a-diazacyclopenta[cd]azulene ***(14)***

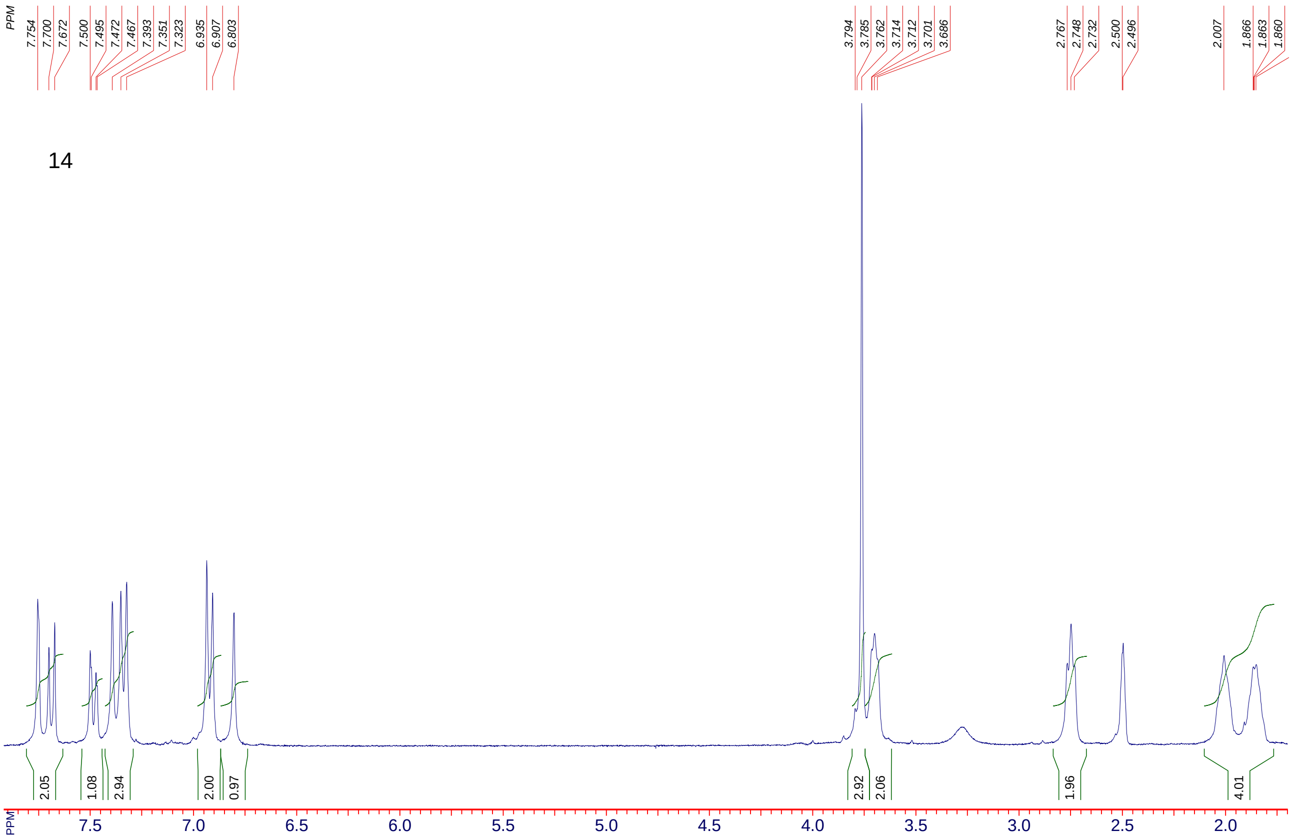

**File name: 14**	Operator:	SF: 299.9450 MHz	NSC: -1	PW: 7.00 usec, RG: 0	SI: 16384
Date:	Solvent:	SW: 5099 Hz	TE: 0 K	AQ: 3.21 sec, RD: 1.50 sec	
